# ManyBabies 3: A Multi‐Lab Study of Infant Algebraic Rule Learning

**DOI:** 10.1111/desc.70259

**Published:** 2026-09-15

**Authors:** Ingmar Visser, Andreea Geambasu, George Kachergis, Cátia M. Oliveira, Joscelin Rocha‐Hidalgo, Martin Zettersten, Teruni Ahamat, Nicolás Alessandroni, Nadja Althaus, Sudha Arunachalam, Suzanne Aussems, Emma L Axelsson, Zeynep Aydin, Heidi A. Baumgartner, Christina Bergmann, Roberta Bettoni, Alexis K. Black, Hermann Bulf, Krista Byers‐Heinlein, Chiara Capparini, Lily Carroll, Alexandra Carstensen, Rebekka Cebulla, Xiaoyun Chen, Kateřina Chládková, Benedetta Colavolpe, Rodica R Constantine, Elísabet Eir Cortes, Frances L. Doyle, Anna Exner, Samuel H Forbes, Laura Franchin, Alessandra Geraci, Judit Gervain, Nayeli Gonzalez‐Gomez, Silje Hagelund, Erin E Hannon, Naomi Havron, Sagi Jaffe‐Dax, Scott P Johnson, Wiktoria Jędryczka, Natalia Kartushina, Melissa Kline Struhl, Ada Koleini, Abhinav Kona, Shannon P Kong, Jessica E Kosie, Monika Kuc̆erová, Katerina Cook, Jared Wayne Leslie, Casey Lew‐Williams, Scarlet Wan Yee Li, Romi Livne, Ellen Marklund, Julien Mayor, Michal Misiak, David Moreau, Jutta L. Mueller, Amber Muhinyi, Alyssa A Quinn, Maartje E. J. Raijmakers, Audun Rosslund, Iris‐Corinna Schwarz, Jeanne L Shinskey, Mohinish Shukla, Sylvain Sirois, Piotr Sorokowski, Basma Sowan, Tibor Tauzin, Angeline Sin Mei Tsui, Martina Turconi, Rebeca C Urizar, Lisa Wagner, Stephanie Wermelinger, Gert Westermann, Ivonne Weyers, Luiza S. B. Yuan, Martina S. Zaharieva, Tania S. Zamuner, Barbara Zapiór, Simone Zasso, J. Grace Zhou, Melanie Soderstrom, Clara C. Levelt

**Affiliations:** ^1^ Department of Psychology University of Amsterdam Amsterdam the Netherlands; ^2^ Leiden University Centre for Linguistics Leiden University Leiden the Netherlands; ^3^ Department of Psychology Stanford University Stanford California USA; ^4^ School of Psychology Cardiff University Cardiff UK; ^5^ Psychology Department University of York York UK; ^6^ Department of Psychology Pennsylvania State University University Park Pennsylvania USA; ^7^ Department of Cognitive Science University of California San Diego La Jolla California USA; ^8^ Department of Psychology Princeton University Princeton New Jersey USA; ^9^ Department of Psychology University of Warwick Coventry UK; ^10^ Department of Psychology Concordia University Montréal Canada; ^11^ School of Psychology University of East Anglia Norwich UK; ^12^ Department of Communicative Sciences and Disorders New York University New York USA; ^13^ School of Psychological Sciences University of Newcastle Newcastle Australia; ^14^ Department of Developmental and Social Psychology University of Padua Padua Italy; ^15^ Center for Neuroscience, School of Advanced Studies University of Camerino Camerino Italy; ^16^ Osnabrück University of Applied Sciences Osnabrück Germany; ^17^ Max Planck Institute for Psycholinguistics Nijmegen the Netherlands; ^18^ Department of Psychology University of Milano‐Bicocca Milan Italy; ^19^ School of Audiology and Speech Sciences University of British Columbia British Columbia Canada; ^20^ Center for Research in Cognition & Neurosciences (CRCN) Université Libre de Bruxelles Brussels Belgium; ^21^ School of Social and Behavioral Sciences Arizona State University Phoenix Arizona USA; ^22^ Department of Psychology Lancaster University Lancaster UK; ^23^ Institute of Psychology Czech Academy of Sciences Prague Czechia; ^24^ Faculty of Arts Charles University Prague Czechia; ^25^ Department of Psychology University of Padua Padua Italy; ^26^ Department of Psychology University of Nevada Las Vegas Las Vegas Nevada USA; ^27^ Department of Linguistics Stockholm University Stockholm Sweden; ^28^ Lifespan Health & Wellbeing Research Centre, School of Psychological Sciences, Faculty of Medicine, Health and Human Sciences Macquarie University Sydney Australia; ^29^ Faculty of Psychology Ruhr University Bochum Bochum Germany; ^30^ Department of Psychology Durham University Durham UK; ^31^ Department of Psychology and Cognitive Science University of Trento Trento Italy; ^32^ Department of Educational Sciences University of Catania Catania Italy; ^33^ CNRS Paris France; ^34^ Centre for Psychological Research Oxford Brookes University Oxford UK; ^35^ Centre for Research on Equality in Education University of Oslo Oslo Norway; ^36^ School of Psychological Sciences University of Haifa Haifa Israel; ^37^ Institute of Information Processing and Decision Making University of Haifa Haifa Israel; ^38^ The Center for the Study of Child Development University of Haifa Haifa Israel; ^39^ School of Psychological Sciences Tel Aviv University Tel Aviv Israel; ^40^ Sagol School of Neuroscience Tel Aviv University Tel Aviv Israel; ^41^ Department of Psychology University of California Los Angeles California USA; ^42^ Institute of Psychology University of Wrocław Wrocław Poland; ^43^ Department of Linguistics and Scandinavian Studies University of Oslo Oslo Norway; ^44^ Department of Brain & Cognitive Sciences Massachusetts Institute of Technology Cambridge Massachusetts USA; ^45^ Children Helping Science Cambridge Massachusetts USA; ^46^ Department of Psychology University of Oslo Oslo Norway; ^47^ Department of BioSciences Rice University Houston Texas USA; ^48^ Department of Linguistics University of Ottawa Ottawa Canada; ^49^ IDN Being Human, Institute of Psychology University of Wroclaw Wroclaw Poland; ^50^ School of Anthropology & Museum Ethnography University of Oxford Oxford UK; ^51^ School of Psychology University of Auckland Auckland New Zealand; ^52^ Centre for Brain Research University of Auckland Auckland New Zealand; ^53^ Department of Linguistics University of Vienna Vienna Austria; ^54^ Department of Psychology, Royal Holloway University of London Egham UK; ^55^ Department of Special Education Stockholm University Stockholm Sweden; ^56^ Département de Psychologie Université du Québec à Trois‐Rivières Trois‐Rivières Canada; ^57^ Department of Psychology University of Manitoba Winnipeg Canada; ^58^ Jacobs Center for Productive Youth Development University of Zurich Zurich Switzerland; ^59^ Department of Psychology University of Zurich Zurich Switzerland; ^60^ Institute for Logic, Language, and Computation University of Amsterdam Amsterdam Netherlands; ^61^ School of Psychology University of Ottawa Ottawa Canada; ^62^ Department of Pediatrics and Child Health University of Manitoba Winnipeg Canada; ^63^ Leiden Institute for Brain and Cognition Leiden the Netherlands

**Keywords:** infants, multi‐lab replication, rule learning

## Abstract

The ability to learn and apply rules lies at the heart of cognition. In a seminal study, Marcus et al. (1999) reported that 7‐month‐old infants learned abstract rules over syllable sequences and were able to generalize those rules to novel syllable sequences. Dozens of studies have since replicated that finding and extended it using different rules, modalities, stimuli, participants (human adults and non‐human animals) and experimental procedures. Yet questions remain about the generalizability of the core findings in Marcus et al., and sources of variation across these findings. In the current study, we address this issue by testing 839 infants (650 after exclusions) of a wide age range (5;0–12;0 months) in a multi‐laboratory (30 laboratories, [31 samples]) conceptual replication of the Marcus et al. study. This study and the analysis plan were submitted as a registered report. A mixed‐model analysis of the looking times at consistent versus inconsistent test trials indicated no significant effect. The current study therefore finds no evidence for rule‐learning; to the contrary, a Bayesian analysis of the data indicated strong evidence for a null effect. This was similarly the case for the moderating factors that were part of the design: mono‐ versus multilingualism, age, and experimental paradigm; none of these factors showed significant effects. Robustness analyses indicated that the results were not different in the case of different choices in the (pre‐)processing of the data. A follow‐up analysis for single labs indicated that there was no evidence of rule‐learning in 30 of the 31 contributed samples. The current finding raises fundamental issues about rule‐learning that are presented in the Discussion section.

## 1 Introduction

Rules are essential in many cognitive abilities, in areas as diverse as problem solving (Anderson 1996), social cognition (Kunkel 1997), cognitive development (Siegler 1983), and developing causal reasoning (Kuhn 2012); the notion of rules and rule learning abilities are core concepts in building domain‐general cognitive architectures (Anderson 1996; Laird 2012). Manipulating cognitive representations by (symbolic) rules has been argued to be required to explain the powerful cognitive abilities that humans have in several areas, such as playing chess, solving reasoning problems, and doing mathematics (Chomsky 1980; Fodor 1981; Fodor and Pylyshyn 1988; Pylyshyn 1984). Finding out if, when, and how rule learning initially develops informs fundamental debates about the architecture and development of the human mind (Marcus 2001; Pothos 2005), as well as comparisons of human cognitive abilities with non‐human species (Fitch 2014; Ten Cate 2017).

Language is one area of cognition that has received a lot of attention with respect to the role of rules and rule learning. Acquiring a human language requires the ability to discover the meaningful units in the surrounding linguistic input (words, morphemes, multiword units), as well as the ability to discover and generalize rules combining these meaningful units at multiple levels (e.g., syntax, phonology, semantics). It has been shown that infants make use of statistical learning mechanisms, specifically to discover units in continuous speech (Maye et al. 2002; Saffran et al. 1996; for an overview, see Saffran and Kirkham 2018; see Cristia 2018, for a critical meta‐analysis of paradigms in statistical learning). However, in their seminal study, Marcus et al. (1999) addressed whether infants also employed a rule learning mechanism that could induce and generalize rules beyond the original stimulus material presented to them, and independent of surface features. Marcus et al. (1999) described this as “algebraic” rule learning (henceforth simply “rule learning”), because the learner must make use of open‐ended abstract relationships or variables that can be expressed with arbitrary items. A principal theoretical impetus for these rule learning experiments was the claim that certain types of neural network architectures, like the simple recurrent network (e.g., Elman 1991), are unable to learn these types of patterns because the networks cannot represent variables and relations between those variables, and thus they cannot generalize outside of their training sample (Marcus 2000).

In the Marcus et al. (1999) study, 7‐month‐old infants were familiarized with a two‐minute stream of speech syllable triplets following either an ABB (e.g., *ga ti ti*) or ABA (e.g., *ga ti ga*) pattern. Following familiarization, infants generalized these patterns to sequences of novel syllables (e.g., *wo fe fe* for ABB and *wo fe wo* for ABA patterns) and showed a novelty response to violation of these patterns (e.g., *wo fe wo* for infants who were familiarized with *ga ti ti*). The authors concluded that infants had learned the abstract rules governing the structure of the familiarized sequence and argued that, in addition to a tool for tracking transitional probabilities (statistical learning), infants also possess a tool for manipulating variables (rule learning) for “learning about the world and attacking the problem of learning language” (Marcus et al. 1999, 79). Many studies have since followed this paradigm to answer questions about whether such rule‐learning ability is specific to linguistic inputs, whether it is even specific to humans, and what the nature of the rule learning mechanism is (for a recent meta‐analysis, see Rabagliati et al. 2019). An outcome of this body of work is that it remains unclear how the rule‐learning effect varies across variables such as age, language background, or methodology. The primary aim of the ManyBabies 3 (MB3) project is therefore to measure the rule learning effect in a consensus paradigm, and examine sources of variation by studying it in a large and diverse sample of infants across many laboratories. In our study we focus on infants' learning of ABA versus ABB patterns—the motivation for this choice is discussed below. Below, we first briefly describe in some detail the classical result by Marcus et al. (1999). Next, we address the motivation for studying rule learning with a large and diverse multi‐laboratory approach, and outline the design choices that were made in the current study.

**Summary**

- Our study provides a large‐scale (30 labs; 839 participants; 650 included in the main analysis), cross‐linguistic, conceptual replication of the seminal study of Marcus et al. (1999).
- A phonologically balanced and broadly interpretable set of stimuli was developed to assess the robustness of algebraic rule learning in infants from multiple language backgrounds.
- Results indicate evidence for a null effect of consistent versus inconsistent trials, showing no evidence for rule‐learning.
- Robustness analyses indicated that our results are robust vis‐á‐vis a number of choices in pre‐processing and analysis.

### 1.1 Rule Learning

In Marcus et al. (1999), rule learning was examined in three experiments. In the first experiment, 7‐month‐old infants were familiarized with 16 different three‐syllable sequences that followed either an ABA or ABB rule. They were then tested on both ABA and ABB test sequences composed of novel syllables. Using the headturn preference procedure (HPP), 15 out of 16 infants showed a novelty preference; on average, infants listened to the inconsistent sequences 40% longer than to consistent sequences (Hedges' *g* = 1.13; this effect size and subsequently reported effect sizes from Marcus et al. (1999) were retrieved from http://metalab.stanford.edu/dataset/rulelearning/). In their second experiment, Marcus et al. (1999) further controlled for a possible confound stemming from the relationship between the patterns of stimulus sequences and the phonetic features of the syllables (e.g., ABA patterns in the first experiment turned out to have voiced‐unvoiced‐voiced consonants in the syllable sequences). Consistent with earlier results, the authors found that 15 out of 16 infants showed a novelty preference for the inconsistent items in the test phase and the overall effect size remained large (Hedges' *g* = 0.76). Finally, Marcus et al. (1999) examined the possibility that infants' successful discrimination of the patterns arose merely from one pattern – ABB – containing a direct repetition of syllables whereas the other – ABA – did not. If this possibility were true, then infants could have just focused on BB versus BA in order to discriminate the patterns, obviating the necessity to learn the algebraic rule. In the third experiment, the authors therefore presented a direct repetition in both conditions, by familiarizing infants with either AAB or ABB sequences and testing them using both AAB and ABB sequences. Again, it was found that infants (16 out of 16) listened longer to the inconsistent patterns in comparison to the consistent patterns with a similar effect size as reported in Experiments 1 and 2 (Hedges' *g* = 1.19). These findings suggested that infants focused on more than just a direct repetition of syllables. In summary, across three experiments, Marcus et al. (1999) reported that 7‐month‐old infants could extract and generalize simple abstract rules from syllable sequences, and that this ability did not appear to be driven by the phonetic make‐up of the sequences or the mere presence of a direct repetition in the syllable sequences.

### 1.2 Multi‐Lab Replication of Rule Learning

This classical result by Marcus et al. (1999) has sparked dozens of follow‐up studies and their paper has been cited over 1000 times. Further, it is frequently mentioned in textbooks on language acquisition that 7‐month‐old infants have rule learning abilities (Gleason and Ratner 2016; Hoff 2014; Kail and Fayol 2015; Lust 2006; O'Grady 2005; Saxton 2010; Sun 2008). Thus, it appears that rule learning is a cornerstone finding in the infant cognition literature.

In order to test whether the ability of rule extraction and generalization is domain‐general (cf. Berent et al. 2021), subsequent studies have extended the rule learning paradigm to different modalities. Overall, these findings suggest that, although infants may show more robust rule learning in the context of speech, they are nevertheless capable of extracting and generalizing simple rules from visual stimuli (e.g., Ferguson et al. 2018; Johnson et al. 2009), from non‐linguistic sound stimuli such as tones or animal sounds (Marcus et al. 2007), and from bimodal visual‐auditory stimuli (e.g., Frank et al. 2009; Tsui et al. 2016). In a meta‐analysis, Rabagliati et al. (2019) reported an average significant novelty effect of Hedges' *g* equal to 0.25 across 91 studies, suggesting that infants demonstrate rule learning across modalities. Note that this average effect size was much smaller than those reported for linguistic stimuli in Marcus et al. (1999). Importantly, the Rabagliati et al. (2019) meta‐analysis showed that there is large heterogeneity in looking time preferences across studies, ranging from large novelty preferences to even large familiarity preferences. Rabagliati et al. (2019) further found that meaningful and speech‐like stimuli yielded the largest effect of infant rule learning studies—around 0.4—, but they also found very high residual heterogeneity in the meta‐regression models, even when other variables were controlled for (e.g., infants' age, experimental design factors). Generally speaking, however, meta‐analyses have been shown to find significantly larger effect sizes than pre‐registered multi‐lab replications (Kvarven et al. 2020). Thus, a precise estimate of the rule learning effect size and how and why infants' rule learning varied across studies remains largely unknown. The current focus is on the most basic and relevant factors that could potentially contribute to heterogeneity: age, linguistic background, and experimental paradigm. Only a large multi‐lab approach can reliably address the issue of heterogeneity across these factors.

Studies examining infant rule learning have mainly come from well‐resourced laboratories in Western societies (Rabagliati et al. 2019), although there are a few notable exceptions, (Tseng et al. 2018; i.e., a few studies in Asia, such as Tsui et al. 2016). This raises concerns about whether infant rule learning (as described in the Marcus et al. 1999) paradigm) is a universal ability that can be generalized to different infant populations across the diversity of human infant experiences. Although we do not have any a priori reason to believe that this ability would vary across populations, it is nonetheless important to increase the diversity of our samples, particularly for effects claimed to be universal.

The current ManyBabies study builds on existing findings in other ManyBabies studies. The first ManyBabies project – MB1 – investigated infants' preference for infant‐directed speech (IDS) over adult‐directed speech (ADS). It generated a number of insights regarding the influence of so‐called “laboratory factors,” participant characteristics, and methodological approaches on the strength of infant looking time effect sizes (Consortium 2020). However, as the first study of its kind, it is not clear to what extent these findings would generalize to other research questions and procedural details. In MB1, the strongest effect size was found using the “Headturn Preference Procedure” (HPP) where infants must make a clear head turn toward the stimulus, compared to approaches that required only eye movement responses, that is, in central fixation (CF) and eye‐tracking (ET). However, there are a number of possible explanations for the higher effect sizes in HPP, some of which could generalize to other studies (e.g., that HPP involves a more active response from the infant and is therefore more sensitive), while others would not (e.g., a correlation between the particular skill set of the specific laboratories using HPP and the question being tested). The methods in the current ManyBabies study differ from MB1 in important ways (e.g., the stimuli used in the current study were designed to be less sensitive to infant's own linguistic experience than those of MB1) and as such allow us to begin to test the generalizability of methodological findings across different paradigms that test the same phenomenon.

In summary, we aimed to carry out a large‐scale replication study to examine infant rule learning across a wide age range (i.e., 5;0 to 12;0 months) and with populations from different language backgrounds, in order to (a) provide a large‐scale conceptual replication of the central rule learning effect, (b) identify factors that may influence this effect, and (c) test the generalizability of the findings across diverse populations.

The design for this study was pre‐registered as Stage 1 Registered report at Developmental Science. The Stage 1 report that received in principle acceptance can be found at the MB3‐OSF repository (https://osf.io/preprints/psyarxiv/aex7v_v1).

### 1.3 Current Study Design

The original motivation for Marcus et al. (1999) to examine rule learning was to understand the computational bases of grammatical constructions that appear to operate over variables such as “verb phrase.” To this end, their stimuli were language‐like and consisted of “words” (speech syllables, created by a Bell‐Labs synthesizer) arranged in “sentences” (trisyllabic sequences). As in the original study, we too employ language‐like stimuli. There are several reasons for this. First, we aimed to keep close to the original study (except where we judged it necessary to deviate, as detailed in the stimuli section). Second, past studies have revealed that 7‐month‐olds were better able to extract rules from sequences of both speech and nonspeech sequences (e.g., musical tones, animal sounds, or varying timbres) during the test phase if they had been familiarized with those rules in sequences of speech (Marcus et al. 2007). Third, the meta‐analysis of Rabagliati et al. (2019) showed that speech‐like stimuli yielded the largest effect of infant rule learning. Another key reason for using language‐like stimuli is that much of the rule learning literature informs debates in language learning, and hence using speech stimuli is important when adding to these debates.

As for the specific rule to learn, studies of infant rule learning have produced mixed results with respect to the role of adjacency in the learnability of a simple abstract repetition rule (Schonberg et al. 2018). In experiments with abstract visual stimuli, direct repetitions seemed to facilitate rule learning such that some rules (e.g., ABB) were easier to learn than others (e.g., ABA; Johnson et al. 2009). Age and the position of the repetition both played a role here too: in Johnson et al. (2009), 8‐month‐olds could only learn from ABB visual sequences, while 11‐month‐olds could also learn from AAB sequences. Neither age group could learn from ABA sequences. However, with pictures of dogs and cats, 7‐month‐olds could learn from both ABA and ABB sequences (Saffran et al. 2007). In auditory rule learning studies, there were mixed results as well. In Marcus et al. (1999), 7‐month‐olds familiarized with ABA sequences of syllables generalized to new syllables when contrasted with ABB, and vice‐versa; that is, infants who learned ABA appeared to recognize this pattern in new items, and identified ABB as a novel pattern. This was also found in (Gerken 2006) with 9‐month‐olds, in Marcus et al. (2007) with 7.5‐month‐olds, and in Thiessen (2012) with infants between 6.5 and 8.0 months old. However, newborns only responded to the difference between ABB and ABC sequences, not to the difference between ABA and ABC sequences (Gervain et al. 2008). Moreover, in a complex hierarchical task where the As and Bs in ABA and AAB patterns consisted of further ABA or AAB patterns, 7‐month‐olds could only learn AAB rules, not ABA rules (Kovács and Endress 2014). When 12‐month‐olds were tested on learning both ABA and AAB rules simultaneously, monolinguals could only learn AAB, not ABA, but bilinguals could learn both (Kovács and Mehler 2009b). There thus appears to be a facilitating effect of direct repetition in previous rule learning studies. In addition to this, it appears that humans, and some other animal species too, are sensitive to repetition and it has been suggested that there is a specialized primitive devoted to processing identity relations (Endress et al. 2009). While both ABB and ABA are examples of repetition patterns, this primitive appears to be especially sensitive to the processing of direct repetitions as in AAB or ABB. In order to be able to find out more about the role of direct versus non‐adjacent repetition in rule learning and given above mentioned previous findings in rule learning, we decided to focus on replicating Experiment 2 of Marcus et al. (1999), where ABA versus ABB is tested, across age and mono‐/multilingualism using a relatively easy comparison of strings that can be distinguished by the presence of either direct or non‐adjacent repetition.

Following MB1, laboratories employed either the HPP, or single screen setups with the CF paradigm or ET to study the magnitude of the learning effect in each of these paradigms. This ensured that we could maximize the number of laboratories able to contribute data in the present study. Within these paradigms, we minimized procedural flexibility wherever possible, while recognizing that laboratories vary in what is technically possible to implement. See the Procedure Section for discussion and details.^1^

Although the meta‐analysis by Rabagliati et al. (2019) investigated the age factor and did not find an age effect, the large majority of rule learning studies have focused on testing 7‐month‐old infants, with only some exceptions (Dawson and Gerken 2009; Frank et al. 2009; Gerken 2006; Gervain et al. 2008; Johnson et al. 2009; Schonberg et al. 2018). As a result, it has been difficult to establish a potential impact of age on the rule learning effect. In this study, we test infants between 5;0 and 12;0 months of age making it possible to investigate the developmental trajectory of rule learning across the entire age range rule learning has been studied in. Our findings in this respect could also help inform a larger question in developmental research, namely whether rule learning develops with age. Interestingly, MB1 found an increase in effect size in the preference for IDS over development. This increase could be due to a developmental change specifically in infants' preference for IDS, but could also be due to an age effect that is more widespread across experimental contexts (Bergmann et al. 2018; Consortium 2020). In the latter case, the present study may be able to capture a more general effect of more robust behavioral responding.

As there are several important reasons for deviating from the original Marcus et al. (1999) paradigm (as described in more detail below), this ManyBabies study should be viewed as a conceptual, rather than a direct, replication. A study recently conducted in The Netherlands (with overlap in authorship with the current study) that exactly replicated Experiment 2 of Marcus et al. (1999) using the original stimuli, paradigm (headturn preference paradigm), and age group (7‐month‐olds) has shown no evidence of replicating the original results (Geambaşu et al. 2023). The lack of evidence for rule learning in this cohort of 96 infants tested across four labs highlights the need for further understanding the factors that may contribute to this phenomenon. The present study is a complementary initiative, and is needed in order to answer remaining questions related to the roles of age, paradigm, and potential role of linguistic background which could not be explored in the Dutch labs.

### 1.4 Research Questions and Hypotheses

The rule learning “effect” is defined as infants' ability to generalize an abstract pattern, operationalized as a post‐familiarization preference (via headturn or visual fixation) for a novel pattern (Marcus et al. 1999; Rabagliati et al. 2019).

Our primary research questions are the following:

1. What is the magnitude and the variability of the rule learning effect in infancy?
2. Is the rule learning effect modulated by age, linguistic background, or experimental paradigm (e.g., HPP vs. CF)?
3. Is rule learning facilitated by direct repetition?

#### 1.4.1 Question 1: What Is the Magnitude and the Variability of the Rule Learning Effect in Infancy?

In MB3 (as in MB1), we employ a consistent set of stimuli across labs to reduce the likelihood that stimulus parameters account for any observed differences in the effect across sites. These stimuli were designed to be appropriate for use across languages with diverse phonologies. In a previous meta‐analysis, large heterogeneity of effects was found across labs, which in part may have been the result of differing stimulus materials (Rabagliati et al. 2019). This possible confound is eliminated here by standardizing the experiment materials. Eventual heterogeneity beyond what can be expected due to sampling error in our findings could hence be attributed to either of two main factors: (1) differences between labs that are not captured in the specification of the experiment's procedures, or (2) heterogeneity in the rule learning effect itself, that is, it being a highly variable effect within and between infants resulting in high sample‐to‐sample differences in its magnitude. If indeed heterogeneity is limited after controlling for testing paradigm, linguistic background, and age, this would imply that rule learning is a robust phenomenon. Finding relatively larger heterogeneity, in contrast, would imply that rule learning is not a robust ability, or that our ability to measure it is influenced by factors outside those we aimed to control. A third possibility is that the magnitude of the effect varies in a systematic fashion according to testing paradigm, language background, or age, as described below. Such a finding could eventually shed light on the nature of the mechanisms underlying rule learning. Related to this first question the research hypotheses are:

**Hypothesis 1a**. *Rule learning is evidenced by a novelty preference for inconsistent patterns at test*.

**Hypothesis 1b**. *After controlling for age, linguistic background, and experimental paradigm, there is little residual heterogeneity between labs*.

Importantly, beyond testing these hypotheses, it is informative to have a robust estimate of the overall effect size as well as an estimate of residual heterogeneity should it be present.

#### 1.4.2 Question 2: Is the Rule Learning Effect Modulated by Age, Linguistic Background or Experimental Paradigm (HPP/CF/ET)?

First, there are not many studies that have extensively reported on the effect of age on rule learning. Generally, it is reasonable to expect that older infants outperform younger infants as the ability to process information develops, in which case the overall effect strengthens with age. In visual rule learning, the effect appeared to increase with age (Johnson et al. 2009; Schonberg et al. 2018). Comparatively, Dawson and Gerken (2009) found that rule learning weakened between 4 and 7 months when musical chords or tones were used as stimuli. Further, the meta‐analysis of Rabagliati et al. (2019) revealed a slight, nonsignificant decline in performance with age. These mixed findings may be related to the use of stimuli from different domains, but this also calls for investigating the effect of age on rule learning in a large‐scale replication study. In the current study, we expect that older infants perform better than younger infants in the rule learning experiment, for two reasons: Rabagliati et al.'s (2019) meta‐analysis has suggested that meaningful stimuli improved infants' rule learning, thus we expect our stimuli (language stimuli) to have the same positive effect, as language is meaningful to all infants and their language ability increases; in addition, since MB1 found evidence of an increase in language related processing skills with age, we expect to find such an effect here too.

Second, we investigate whether the rule learning effect varies by language background. Our sample provides a more diverse representation of languages compared with prior studies and as such speaks to the generalizability of the rule learning finding. However, because we do not have any specific hypotheses regarding cross‐language differences in rule learning, and because variation in language is highly confounded with laboratory‐based differences, we did not directly test this question. There are more compelling and testable reasons to believe that infants may perform differently based on the number of languages they are exposed to. Some studies have suggested that infants from multilingual households outperform infants from monolingual environments in rule learning (Kovács and Mehler 2009a, 2009b) due to enhanced experience and efficiency with “task switching,” or shifting between dimensions and sets (Comishen et al. 2019; Kovács and Mehler 2009a, 2009b; Pour Iliaei et al. 2021; Prior and MacWhinney 2010). However, other studies have found little evidence for a bilingual advantage in switching or rule learning in infants (e.g., D'Souza et al. 2020; Kalashnikova et al. 2021; Tsui and Fennell 2019). We therefore do not have a specific prediction regarding whether a bi‐/multilingual rule learning advantage in infants younger than 12 months is present, but because both monolingual and bi‐ or multilingual infants participated, our study provides information on this issue.

Third, we ask whether the rule learning effect is modulated by the testing paradigm. In particular, we test the hypothesis (given the findings of MB1) that HPP offers a better or more reliable assessment of infant rule learning than methods using eye movements in response to a single screen (i.e., CF and ET) as the dependent variable. While this study on its own cannot rule out potential confounds such as idiosyncratic differences in laboratory experiences with methods or populations, it provides important data regarding the robustness of the stronger effect in HPP found in MB1.

An important auxiliary hypothesis is that there is habituation at test, and that this habituation occurs more strongly in older infants than in younger infants (Hunter and Ames 1988). This results in decreasing looking times on subsequent test trials with larger decreases in older infants. In sum, hence, the hypotheses related to our second research question are:

**Hypothesis 2a**. *Rule learning is robust across age and increases with age, indexed by increased novelty preference for inconsistent patterns at test*.

**Hypothesis 2b**. *Looking times decrease with test trial; this effect is stronger for older infants than younger infants*.

**Hypothesis 2c**. *The relationship between multilingualism and rule learning is explored. Both absence or presence of this effect or its reverse can inform the debate about potential cognitive advantages related to multilingualism*.

**Hypothesis 2d**. *Rule learning is robust across variation in experimental paradigms and is larger in the HPP relative to other paradigms*.

#### 1.4.3 Question 3: Is Rule Learning Facilitated by Direct Repetition?

From the literature discussed above it appears that under more difficult circumstances, that is, when rules have to be learned from abstract visual stimuli (Johnson et al. 2009), from more complex stimuli (Kovács and Endress 2014) or when two rules have to be learned simultaneously (Kovács and Mehler 2009b), infants are not able to learn repetition patterns when they involve non‐adjacent items (ABA). Furthermore, neonates only seem to be sensitive to syllable sequences with adjacent repetitions, that is AAB and ABB (Gervain et al. 2008). Finally, in a study by Geambaşu (2018) 7‐month‐old infants were more interested in AAB test items than in ABA test items, independent of their training sequence (AAB or ABA). By studying the rule learning abilities of a large number of infants, in a wide age‐range and with different linguistic backgrounds, we can seize the opportunity to analyze the effect of this factor. The hypotheses related to our third research question are:

**Hypothesis 3a**. *There is an overall preference, that is, longer looking times, for adjacent repetition items (i.e., ABB items) in the test phase, regardless of training condition*.

**Hypothesis 3b**. *There is a larger effect of learning overall in the participants trained on adjacent repetition items (i.e., ABB) than the participants trained on other items (i.e., ABA)*.

## 2 Methods

### 2.1 Participants

Participating laboratories were recruited via a call for participation through social media, relevant listservs, and by word of mouth. To confirm their intention to participate, laboratories completed a registration form prior to data collection that included information about laboratory characteristics, population characteristics, laboratory‐specific protocol details and information relevant to preregistration (e.g., sample size commitment), and the research paradigm they would use. Laboratories were responsible for obtaining any necessary ethics approval via the ethics oversight committees at their home institutions. In locations where no such oversight exists, laboratories were given the option to obtain ethics approval either as a rider to an existing approval from another laboratory or submit a signed statement committing to conducting their research in accordance with established research ethical norms, including but not limited to the Declaration of Helsinki. Laboratories committed to a minimum sample of *n* = 16 participants with usable data, but data from laboratories that were unable to meet this minimum sample were nonetheless included. Data collection took place between January 2023 and March 2024.

A total of 30 labs contributed data. One lab contributed 2 samples with different methods (HPP & ET), resulting in a total of 31 samples; one lab contributed a sample with unusable data—due to a technical error the familiarization time was double the time described in the protocol (i.e., 4 instead of 2 min; these data are analyzed in the exploratory results section). Table 1 provides details regarding the participating laboratories, with 6 laboratories using the HPP (of which 1 used offline coding and the rest used online coding), 14 laboratories using an eye‐tracker (all automatic coding), 11 laboratories using CF (of which 1 used offline coding and the rest used online coding). Note that almost all of participating labs had extensive prior experience with infant testing and with the specific methods applied in this study. The lab manual (https://osf.io/kqu9v/overview) provided further detailed instructions on how to prepare for data collection and the lead team and other labs were available for guidance whenever labs asked for this.

**TABLE 1:** Information for participating labs and demographics.

Lab Id	Country	Most common primary language	*N*	Excl	Mean age (months)	SD (age)	Sex (M/F)	Mean Primary Language Exposure (%)	SD (Exposure)	Testing Method	Coding Method
BiccBabyLab	Italy	Italian	21	6	8.53	2.44	9/12	87.29	18.01	CF	online
LALunipd	Italy	Italian	32	3	9.69	1.95	20/12	94.78	11.58	HPP	offline
StockholmBL	Sweden	Swedish	21	5	8.99	2.01	6/15	87.38	16.33	CF	online
UNLVmusiclab	United States	English	17	2	9.27	1.94	5/12	100.00	0.00	CF	online
UPFbabylab	Spain	Spanish	20	3	8.71	0.65	12/8	78.30	18.64	HPP	online
babellabVienna	Austria	German	40	11	9.00	0.98	21/19	90.22	16.74	ET	automatic
babylabAmsterdam	The Netherlands	Dutch	41	26	9.12	2.28	28/13	72.73	19.87	HPP	online
babylabAmsterdam	The Netherlands	Dutch	16	3	10.53	2.07	9/7	77.00	17.24	ET	automatic
babylabBrookes	United Kingdom	English	39	5	8.70	2.28	21/18	96.69	10.76	CF	online
babylabPrinceton	United States	English	22	2	8.95	1.73	9/13	89.18	14.45	HPP	online
babylabTrento	Italy	Italian	16	2	8.75	1.76	8/8	98.00	5.61	ET	automatic
babylabULB	Belgium	French	17	1	9.09	1.75	7/10	82.53	22.01	CF	online
babylab_leiden	The Netherlands	Dutch	33	4	8.67	1.85	20/13	68.61	23.15	HPP	online
babylingOslo	Norway	Norwegian	17	0	8.73	2.18	8/9	92.35	14.70	ET	automatic
beinghumanwroclaw	Poland	Polish	37	2	7.48	1.51	18/19	96.92	9.27	CF	offline
bllumanitoba	Canada	English	35	4	8.22	1.92	15/20	93.77	10.70	HPP	online
cclruottawa	Canada	English	29	5	9.43	2.59	17/12	83.00	18.20	ET	automatic
ddlabUEA	United Kingdom	English	31	5	8.98	1.87	13/16*	98.44	5.18	ET	automatic
epsyRUB	Germany	German	31	3	10.62	1.49	14/17	96.45	10.42	CF	online
gertlabLancaster	United Kingdom	English	57	32	8.11	2.11	33/23*	100.00	0.00	ET	automatic
irlConcordia	Canada	English	19	3	7.65	1.53	11/8	73.95	18.66	ET	automatic
kidsdevuniofnewcastle	Australia	English	16	1	8.72	1.67	9/7	92.12	17.19	ET	automatic
langdevHaifa	Israel	Hebrew	17	4	7.51	2.22	7/10	95.41	9.93	ET	automatic
langdevUBC	Canada	English	11	0	8.77	1.64	6/5	72.27	20.42	ET	automatic
nyulearn	United States	English	17	1	8.73	2.18	10/7	82.88	20.11	CF	online
rohobabylab	United Kingdom	English	29	8	8.19	1.94	13/16	93.03	13.61	CF	online
sociocognitivelab	Norway	Norwegian	11	3	8.93	1.82	6/5	92.73	16.79	ET	automatic
speakinlab	Czech Republic	Czech	33	2	8.61	1.96	18/15	95.47	11.02	CF	online
tauccd	Israel	Hebrew	18	2	9.19	2.09	7/11	90.59	14.78	ET	automatic
warksbabylab	United Kingdom	English	17	0	8.38	2.54	4/13	94.41	13.21	CF	online
weltentdecker‐zurich	Switzerland	German	79	10	8.64	1.91	41/38	83.37	17.44	ET	automatic

*Note*: The participants for speakinlab are not included in the main analyses; their data is used in the exploratory analyses section. *gender information missing for 2 and 1 participant(s), respectively.

Beyond the demographic information in Table 1, the current sample is characterized by a large heterogeneity with participants from 16 different countries, 33 different languages reported as the participants' primary language and a larger age‐span tested than in any other rule‐learning study. Participating labs were all located in urban areas and as a consequence the large majority of participants is expected to reside in urban areas as well (note however that no direct data was collected about this). Labs were encouraged to collect data about the community of descent of their participants when this is deemed appropriate in their context. 17 out of 30 labs did collect either race or ethnicity or both, using category labels that are locally appropriate (61 different labels were used for the race construct). While summarizing these data is very hard, one clear observation stands out: In the labs where race is reported, the majority category is “White” (when including “Caucasian” under “White”) in 11 out of 14 labs. Similar observations hold for the ethnicity variable. Socioeconomic status in our participants shows some heterogeneity although caregiver education is overall (very) high; in all labs a (large) majority of the primary caregivers has enjoyed higher education (bachelor, master, phd or equivalents). The full details of the demographics questionnaires are available through the MB3‐analysis‐public github repository (https://github.com/manybabies/mb3‐analysis‐public/).

#### 2.1.1 Participant Inclusion and Exclusion

Participant recruitment targeted infants from 5;0 months (152 days) to 12;0 months (365 days). There were no specific guidelines for individual laboratories regarding distribution of infants within this range, although laboratories were encouraged to test across as wide an age range as possible. Inclusion criteria for participation were: full term (born at 37 or more weeks gestation), with no parent‐reported developmental disorders or sensory impairments. Physical developmental disorders that do not affect cognition or the infant's ability to respond to the stimuli did not count as exclusion criteria. Each lab provided all data (in anonymized form) collected for the study to the analysis team. Participants' data were excluded from analyses based on the following criteria, applied sequentially:

1. Age. Infants younger than 4;0 months or older than 13;0 months were excluded from analyses.
2. Pre‐term. Pre‐term infants, here defined as fewer than 37 weeks gestation, were excluded from analyses.
3. Diagnosed developmental disorders. Infants were excluded based on parental reports of developmental disorders, vision, or hearing impairments.
4. Session‐level errors. Session‐level errors are errors that impact the entire session, and resulted in excluding a participant's data from analyses completely. Session level errors include:

a. equipment error (e.g., an eye tracker failing to record data),
b. experimenter error (e.g., an experimenter becoming unblinded when measuring infants' looking by live button press), evidence of parent/outside interference (e.g., parents talking or pointing at the screen, loud construction noise, siblings entering the room or pounding on the door).

5. Insufficient usable data, that is, less than one pair of test trials. To be included in the data‐analysis, an infant must have contributed valid looking times on at least one ABA trial and one ABB trial, after trial level exclusions are applied (see Data Analytic Approach section for details). Hence exclusion criteria were:

a. fussiness causing the infant not to reach the test phase,
b. infant fussiness leading to absence of valid test trials,
c. equipment error (e.g., an eye tracker failing to record data).

In addition to the exclusion criteria in the registered report, two additional criteria were applied. First, infants for which the age was a missing variable were excluded—their number is subsumed under the age exclusions in the Table 2. Second, a number of infants were presented with too many (7) of either consistent or inconsistent trials during test—these infants were excluded also (listed separately in the Table).

**TABLE 2:** Number of excluded infants per criterion.

Exclusion criteria	*N* remaining	*N* excluded	Cumulative excluded
Pilot	837	2	2
Age	834	3	5
Preterm	823	11	16
Developmental disorders reported	821	2	18
Marked exclude	719	102	120
‐ Equipment error	719	31	—
‐ Experimenter error	719	40	—
‐ Fussiness causing the infant not to reach the test phase	719	8	—
‐ Infant fussiness leading to absence of valid test trials	719	19	—
‐ Outside interference	719	4	—
Excessive number of trials per condition	713	6	126
Marked trial error	710	3	129
Missing minimum valid trials	681	29	158
Double familiarization time	650	31	189

The number of exclusions resulting from each of these criteria is listed in Table 2. Participating labs contributed data from 839 infants in total. After sequential application of the exclusion criteria, the final sample size for analysis was 650 infants (320 male, 328 female, 2 missing ‐ sex other than male or female was provided as an option but none were reported). Table 2 specifies the number of excluded infants for each of the exclusion criteria. The infants from the lab with double familiarization time are listed separately. This group is excluded from the main analyses, but is analyzed further in the Exploratory analysis section.

### 2.2 Stimuli

The stimuli in the experiment we are replicating, Experiment 2 of Marcus et al. (1999), consisted of synthesized speech syllables in a male voice. In the original article, the syllables used in the familiarization sequences were rendered orthographically as “le” “je” “we” “de” “li” “ji” “wi” and “di” and those in the test as “ba” “po” “ko” and “ga.” In order to understand how these would sound exactly, we asked the first author of the original paper, Gary Marcus, permission to analyze the original stimuli, which could be provided by Scott Johnson. The stimuli were analyzed in Praat (Boersma and Weenink 2019) and transcribed using the International Phonetic Alphabet (IPA)—(see Table 3)—by two of the current authors.

**TABLE 3:** International phonetic alphabet transcripts of the stimuli in Experiment 2 of Marcus et al.

Syllable	Transcript	Syllable	Transcript
“le”	[li]	“wi”	[waı]
“je”	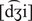	“di”	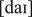
“we”	[wi]	“ba”	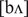
“de”	[di]	“po”	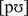
“li”	[li:]	“ko”	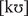
“ji”	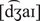	“ga”	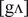

*Note*: IPA transcripts of the stimuli in Experiment 2 of Marcus et al. (1999).

The original syllables turned out to contain several English‐specific sounds, like the affricate 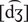 and the vowels 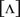 and [aı], and the syllables “li” and “le” sounded very similar to each other. The syllables also had different durations, varying between 216.8 and 415.2 ms, and varying intonation contours.

In the current study, we chose to deviate from the original stimuli in several ways, for a number of reasons. First, because our language stimuli would be presented to infant participants from many different language backgrounds, we opted for a set of less English‐specific sounds that would be perceived as distinct from each other across a diverse set of languages. The specific sounds were chosen, based on The World Atlas of Language Structures Online (ref 2013), to ensure correspondence with distinct phonemes within the sound inventories of the expected set of languages across participating labs, that is, Dutch, English, French, German, Hungarian, Italian, Japanese, Spanish, Hebrew, Arabic, and Swahili. While the chosen set of vowels and consonants are not necessarily perceived in exactly the same way across languages – for example, we included stop consonants with a voice onset time (VOT) of ~0 ms, which is interpreted as unvoiced in languages like Dutch and French but as voiced in languages like English and German – the resulting syllables that make up the ABA and ABB patterns in the experimental sequences should be interpretable and perceived as different from each other by all participants. In addition, we collected information from participating laboratories to confirm that the sounds are discriminable and felicitous across all tested languages. Any cases of concern were examined through robustness testing (see analysis section).

Second, our syllables are based on natural speech — as we expect naturally spoken syllables to facilitate learning as they are more language‐like — spoken by a male voice. At the same time, to avoid confounds triggered by patterns in intonation contours or differences in syllable length, the recorded syllable tokens were subsequently manipulated in Praat (Boersma and Weenink 2019) to create a set of individual syllables that are matched on various prosodic measures (see Syllable Preparation and Validation section below).

The final list of syllables is composed of specific combinations of the consonants [p], [t], [k], [f], [s], [l], [n], [m] and the vowels 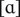, [o], [u], [e], [i], and individual syllables are expected to be neither odd nor unattested in any of the tested populations' languages, even if they might be transcribed differently by speakers of different language backgrounds.

#### 2.2.1 Syllable Preparation and Validation

Stimuli were recorded in a quiet space using a Behringer Ultravoice Xm8500 Dynamic Vocal Cardioid Microphone with a pop shield, onto a Marantz solid state recorder in battery mode. Syllables were produced using as even a rate and as flat a prosody as possible. All subsequent handling and analyses were in Praat.

Individual syllables were manually segmented out, and clipped to nearest zero‐crossings. All syllables were then concatenated with 250 ms silence between each. The entire concatenated sound file was pitch flattened by setting the pitch to a constant value of 132 Hz (the mean pitch across the recorded syllables). Individual syllables were then segmented out again and adjusted to have a duration of 250 ms (actual average 248 ms ± 6.4 ms), intensity of 70 dB (actual average 69.83 dB ± 0.3 dB), and mean f0 of 132 Hz (actual average 132.38 Hz ± 0.55 Hz) each. Pilot transcriptions by members of this author group led to some individual syllable tokens being re‐recorded.

We then gathered syllable identification data for the final set of syllables using Amazon Mechanical Turk. Participants (*N* = 87) judged the identity of each syllable and provided a rating of the prototypicality/quality of the syllable they identified. During the identification task, participants heard a series of 22 trials with a random order of the syllables “fa,” “fe,” “fu,” “ka,” “ke,” “ku,” “la,” “li,” “lo,” “ma,” “me,” “mu,” “ni,” “no,” “pa,” “pe,” “pu,” “si,” “so,” “ta,” “ti,” “to.” They could press the “r” key to repeat the syllable as often as they pleased. Pressing any other key took them to the next screen where they could type the perceived identity of the syllable into a text box. When they hit Enter or clicked Next, they were shown a scale from 1…7, with 1 labeled (Poor example) and 7 labeled (Great example). Typing a number between 1 and 7 recorded their response and moved them to the next trial. Modal responses matched the actual syllable for all tokens, and mean prototypicality/quality ratings were all >4. The stimuli, further details about their creation and the full data of the syllable ratings can be found at the MB3‐OSF repository (https://osf.io/kqu9v/overview).

#### 2.2.2 Training and Test Sequences

The final selection of syllables was divided into training and test sets, ensuring that phonetic features (stops, nasals, open vowels, etc.) were as evenly distributed across training and test sets as possible. The syllables in the training set were constructed with the consonants [p], [k], [f], [m] and the vowels 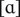, [u], [e], while the syllables in the test set were constructed with the consonants [t], [s], [l], [n] and the vowels [o], [i]. Further, within each group, syllables were assigned to either A or to B classes to enable construction of the ABB/ABA sequences. The full syllable set is shown in Table 4 below.

**TABLE 4:** Syllable sets.

set	syllables
set	syllables
Training A	“ku,” “ke,” “mu,” “ma,” “fe,” “pa”
Training B	“ka,” “me,” “fa,” “fu,” “pe,” “pu”
Test A	“ti,” “lo”
Test B	“so,” “ni”

*Note*: Syllables assigned to A and B classes in training and test.

Individual “sentences” were constructed by concatenating syllables from A and B classes within training‐ and test groups in all possible combinations, for both ABA and ABB structures, with the restriction that the A and B syllables within a given sequence do not share the vowel or the consonant. This restriction prevents the possibility of presenting “XXX” patterns over three identical consonants (e.g., “fefafa”) or vowels (e.g., “pafafa”); hence, both the consonants and the vowels within the sequence highlight the ABB or ABA pattern (Thiessen 2012). This contrasts with the original Marcus et al. (2007) stimuli, in which 7 out of the 16 training sequences contained identical vowels (“le je le” 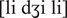, “le we le” [li wi li], “wi di wi” [waı daı waı], “ji di ji” 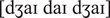, “de je de” 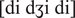, “de li de” [di li: di] “de we de” [di wi di], 3 contained identical consonants (“wi we wi” [waı wi waı], “je ji je” 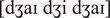, “de di de” [di daı di], and 1 contained both identical consonants and vowels (“le li le” [li li: li]). While we cannot rule out the possibility that some sequences could resemble words in some languages, the pauses in between the individual syllables and the lack of stress and intonation render it less likely that combinations of syllables within the triplets are perceived as word‐like units. Individual syllables that could be meaningful to the participating infants also occurred in the original study, like [dai] “die” [wai] “why” and [wi] “we.”

As in the original stimuli, each trisyllabic sequence has a 250 ms silence between the syllables. Training and test sequences are constructed by concatenating “sentences” of either the pattern ABB or the pattern ABA into pseudo‐random sequences (see Procedure section for details) with a 1.2 s pause inserted between sentences. Table 5 shows the familiarization and test items for both the ABA and ABB patterns.

**TABLE 5:** Familiarization and test sequences.

Familiarization sequences ABA	Familiarization sequences ABB	Test sequences
“fekafe”	“fekaka”	“lonilo”
“fepufe”	“fepupu”	“lonini”
“kefake”	“kefafa”	
“kefuke”	“kefufu”	“tisoti”
“kepuke”	“kepupu”	“tisoso”
“kufaku”	“kufafa”	
“kumeku”	“kumeme”	
“kupeku”	“kupepe”	
“mafuma”	“mafufu”	
“mapema”	“mapepe”	
“mapuma”	“mapupu”	
“mufamu”	“mufafa”	
“mukamu”	“mukaka”	
“mupemu”	“mupepe”	
“pafupa”	“pafufu”	
“pamepa”	“pameme”	

*Note*: Familiarization and test sequences following both the ABB and ABA pattern.

### 2.3 Procedure

The experimental procedure consisted of a familiarization phase followed by a test phase. During the familiarization phase, infants were exposed to a speech stream following either an ABA or an ABB pattern. Either stream was composed of 16 unique sequences which were each presented three times, randomized within blocks of 16 sequences. The streams thus had a duration of approximately two minutes (116.3 s).

The test phase consisted of three blocks of four test items (two different ABA and two different ABB sequences), for a total of 12 test trials, composed of syllables that were not presented in the familiarization phase. Test items are therefore considered either consistent (familiar) or inconsistent (novel) with the pattern presented in the familiarization stream. Per test trial, each sequence was repeated for a maximum of six times with a 1.2 s pause between repetitions, resulting in a trial of a maximum of 15 s in duration. Due to slight variations in syllable duration, triplets and test streams varied slightly in total duration depending on how they were combined. In order to ensure test trial duration of precisely 15 s for all test trials, the final pause was longer than the inter‐triplet pause and varied slightly between concatenated test streams (pause range 1.5–1.8 s). A test trial was valid only with a minimum of 1 s looking time, as that is the minimal time that is required to disambiguate whether an ABA versus an ABB trial is being presented.

#### 2.3.1 HPP

In the HPP testing paradigm, infants were positioned on a caregiver's lap, or near the caregiver in an infant seat, in a quiet room, with the caregiver wearing headphones playing masking auditory stimuli. The HPP setup included a CF point and one or two fixation points on the left and/or on the right side of the infant. The fixation points were either blinking lights or screens, depending on each laboratory's hardware. In the original study, lights were used. Speakers were located in close proximity to the side fixation point(s).

The familiarization phase was initiated with an attention grabber ‐ visual only, no sounds—played from the CF point. Once the infant fixated on the central attention grabber, the familiarization stream began to play, continuously and independently of the infant's attention.

After the familiarization phase, an attention grabber was played to refixate the infant's attention to a neutral central position. Once the infant fixated to the center, the testing phase began.

Each test trial began with the initiation of one of the visual stimuli on either side of the infant. Once the infant turned his or her head to the visual stimulus, the accompanying auditory stimulus began to play. Each test trial played for as long as the infant maintained attention towards the visual stimulus, or until the maximum of 15 s per trial was reached. If the infant looked away for less than 2 s, both the auditory and the visual stimuli continued to play. If the infant looked away for more than 2 s, both the auditory and the visual stimulus ended and thereby the trial would end. Once the test trial had ended, the attention grabber would be played again until the infant fixated on the neutral central point, after which a new test trial would start. This proceeded until all 12 test trials were played.

#### 2.3.2 CF and ET

The CF and ET paradigms proceeded in a similar manner to the HPP. The key difference was that in these paradigms, a single screen was used instead of multiple lights or monitors. In these setups, infants were also positioned on a caregiver's lap or in an infant seat in a quiet room, while parents wore headphones playing masking auditory stimuli. In addition, in the ET set‐up, the look‐coding is typically done using an ET software program (all participating ET labs in fact did this). Note that there are some minor methodological variations from previous rule learning studies using CF and ET in order to more closely parallel those of the HPP.

During the familiarization phase, one of the familiarization streams, following either the ABA or ABB pattern, was played uninterrupted, while the screen showed a static checkerboard pattern over its entirety. This proceeded independent of whether the infant fixated on the visual stimulus or not. Once the familiarization stream was finished, a dynamic attention grabber was played to refixate the infant's gaze. The attention grabber was played until the experimenter or the eye‐tracker software determined that the infant had looked for 500 ms. If the maximum duration (15 s) was reached without a fixation, the attention grabber restarted. Once the infant fixated on the attention grabber, the first test trial was initiated.

For test trials, as in the familiarization phase, the static checkerboard pattern was shown on the entirety of the screen, while the auditory stimulus was playing. If the infant looked away from the screen for less than 2 s, the auditory and visual stimuli continued to play. If the infant looked away from the screen for more than 2 s, both the auditory and the visual stimuli were stopped and the trial would end. Once the test trial ended, the attention grabber stimulus would play again until the infant fixated on it, after which a new test trial began. This proceeded until all 12 test trials were played.

#### 2.3.3 Pilot

A pilot study was conducted by one laboratory in March, 2021 to verify the feasibility of the experiment's design, to test the planned procedures, and to test‐run the planned analysis. The lab used the ET procedure. Given the small size of the pilot, no significant effects were observed, but nonetheless the planned analysis was carried out; the full pilot report can be found at the MB3‐OSF repository (https://osf.io/kqu9v/overview).

The pilot data gathering and the pilot data results resulted in one minor change in the procedure where the length of CF for ET set‐ups was lowered to 500 ms—that is, time prior to the start of the trial that the infant fixates the central stimulus.

### 2.4 Power Analysis

In March 2021, an initial call for participation of laboratories was posted on several mailing lists for developmental and infant researcher communities. As of June 2021, 25 laboratories expressed their interest in contributing with an average of over 20 infants planned to be tested. To be somewhat conservative, our power analysis assumed that a minimum of 20 laboratories would recruit 20 participants (after exclusions), for a total of 400 infants (age range: 5–12 months of age). As traditional power analysis is not possible for mixed‐effects regressions, we instead used a simulation approach, in which we repeatedly generated random datasets (N = 20 laboratories * 20 children * 12 trials = 4800), specifying a chosen effect size (e.g., 0.3 SD, the average effect size across all published developmental experiments; Rabagliati et al., 2019) for any main/interaction effects of interest. For simplicity, we generated datasets using the same specified effect size for trial type, age, and their interaction, and used a slightly simpler regression model than is used in the full analysis: log(looking time) ~ 1 + trial_type * trial_number + trial_type * age + (1|subject) + (1|lab). Power was evaluated by examining in how many of 1000 such generated datasets the mixed‐effects regression estimated a significantly non‐zero (*p* < 0.05) coefficient. We conducted three such power simulations, assuming effect sizes (Cohen's *d*) of 0.1, 0.2, or 0.3^2^ for the three main effects of interest (trial type, age, and trial type * age). For effects of size *d* = 0.1, 92.8% (928 of the 1000) of the simulations recovered a significant trial type coefficient, and 100% of simulations showed a significant age * trial type interaction. For effects of size 0.2 or 0.3, 100% of simulations showed significant trial type effects, as well as age * trial type interactions. Thus, our power analysis suggests ample power to detect the expected trial type effect and expected interactions with age, even if the effect size is quite small (0.1–0.2). A more elaborate description of the power analysis including the R‐code used can be found at the MB3‐OSF repository (https://osf.io/kqu9v/overview).

### 2.5 Data Analytic Approach

Laboratories provided anonymized data for all infants to the analysis team. Exclusions for data analysis on the infant level are detailed in the Participant section. Contributing laboratories were asked to include first‐session infants only where possible; however, any second‐session infants (i.e., infants run in a different study on the same visit prior to their participation in MB3) we received were included in the data, but labeled as such and we planned to analyze the effect of this inclusion in the robustness analyses (see Robustness Analyses section below). We also asked laboratories contributing second‐session infants to document the nature of the study run prior to the current study.

Data from individual trials were discarded in the case of trial‐level errors. Trial‐level errors are errors that affect a single trial, but do not impact the entire session. For example, the experimenter might accidentally end a trial before the infant has finished looking, or a parent might briefly interfere during one trial. These errors would render the specific trial unusable, but are unlikely to affect other trials. In these cases, only the specific trial was classified as an error and excluded from analyses. Looking times were log‐transformed, and trials with zero looking time were excluded from the analyses (Csibra et al. 2016).

The final sample consisted of 650 infants included in the analysis, and, after exclusion of the trial‐level errors, they completed an average of 10.7 trials, out of a total possible of 12. The total number of test trials available for analysis was 6975.

#### 2.5.1 Dependent and Independent Variables

The following variables were included in the analyses (variable names are bolded and, for categorical variables, levels are italicized):

- Infants' looking time (**LT**, log‐transformed) is our dependent variable. Looking time is defined as the time spent looking at the screen (for CF and ET methods and some HPP set‐ups) or at the light (HPP) during test trials.
- **Familiarization_rule** indicates the sequence to which infants were exposed during familiarization (either *ABA* or *ABB*). Infants were exposed to only one sequence, with the sequence determined by random assignment. This variable is a binary variable in which trials presenting infants with the ABA rule were coded as 0.5 and trials presenting infants with the ABB were coded as ‐0.5.
- Trial type (**trial_type**) indicates for each test sequence whether it follows the same rule to which the infant is familiarized or a different rule. For example, if an infant heard an ABA rule during familiarization, ABA test trials were the *same* trial type and ABB test trials were the *different* trial type). This variable is a binary variable in which the same trial type is coded as 0.5 and a different trial type is coded as ‐0.5.
- Test with **repetition** sequence; whether a test trial was *ABB* or *ABA* (but note that this is not the same as trial type, which concerns consistency between familiarization and test trials).
- Trial number (**trial_num**) indicates the sequential order in which test trials are presented. Trial number thus ranges from *1* to *12*, and was centered and standardized before entering the regression.
- Infant **age** indicates the infants' age in months (real‐valued – not rounded). To aid with interpretability, age was centered and standardized before entering the models.
- Experimental **method** indicates the method that was used to record infants' responses to the stimuli, one of: *HPP*, *CF*, or *ET*; we used deviation coding for the **method** variable.
- **Lab** indicates the laboratory in which data were collected. Each laboratory has a unique laboratory identifier.
- **Subject** indicates the unique subject identifier for each infant tested.
- Multilingual exposure (**multilingual_exposure**) indicates the infants' proportion of exposure to other languages than the primary language, with a minimum of 0, meaning no exposure to any but the primary language. Infants with higher scores have more bilingual/multilingual exposure. Multilingual exposure was centered and standardized before entering the models.

Besides the above variables that are part of our planned analyses, a number of other variables were collected that may be useful in later secondary data‐analyses, for example, participant gender and demographic background variables.

#### 2.5.2 Statistical Modeling

To test our hypotheses, we used mixed‐effect models to examine effects of the independent variables (IV) on the dependent variable (DV). Mixed‐effect models allow us to control for random effects in both the DV (“random intercepts”) and the relationship of the IV to the DV (“random slopes”) based on relevant grouping units (subjects and laboratories). We began with a fairly complex model including a maximal random effects structure for the subject level and including the most important lab level random effects (Barr et al. 2013) (listed below). This random effects structure was iteratively pruned after non‐convergence was found, by first removing random effects that were theoretically less important (see the Pruning the Model Section below), and then repeating this procedure by gradually removing other random effects until the model converged. All models were fitted using the lme4 package (Bates et al. 2021) and *p*‐values were computed using the lmerTest package (Kuznetsova et al. 2017). All analyses were run in R (R Core Team 2025).

The mixed‐effects model is specified below in lme4 R code. Using R code, lower‐order effects are subsumed by interactions in the model, even though they are not explicitly written. For example, the interaction between age and trial_type (age * trial_type) enters a main effect of age and a main effect of trial type into the model. Our initial full model structure is:

+ familiarization_rule*trial_type

+ age * trial_type

+ experimental_method * trial_type

+ multilingual_exposure * trial_type

+ trial_num*trial_type

+ trial_num*age

+ repetition*trial_type

+ trial_num*trial_type | subject

+ trial_type + test_order | lab

The model included fixed main effects for familiarization rule, trial type, trial number, infants' age, experimental paradigm, multilingual exposure, and repetition. Main effects tested how the IV affects infants' looking time. For example, trial number models the potential decline of infants' looking time over subsequent test trials, and trial type examines whether infants in the testing phase look differently at the trials that are either congruent or incongruent with the testing rule learned during familiarization. Importantly, we enter a number of two‐way interactions as fixed effects to test our research questions (see details below where we outline which statistical tests relate to which hypothesis).

We account for non‐independence at the subject‐ and laboratory‐levels using the model's random‐effects structure. For the subject‐level grouping, the maximal model estimates random intercepts and random slopes for trial number, trial type, and their interaction, as well as the covariances between random effects (Barr et al. 2013). These effects model the differences in habituation rate across individuals and differences in individuals' preferences for items following the same/different rule at test. For the laboratory‐level grouping in the full model, we include a random intercept, random slopes for test order and trial type, and all covariances.

As some of the tested effects of interest are also informative when they show a null effect we also report Bayes factors alongside the frequentist statistics. Bayesian models were estimated using the brms package in R (Bürkner 2017). Bayesian model specification was identical to the frequentist mixed‐effects models (after pruning). Models were fitted using the default prior specification provided by the brms package, namely flat priors for fixed effects (uniform distribution over all possible parameter values) and student‐t priors (d*f*  = 3, sigma = 2.5) for random effects. Bayes factors for the coefficients of the models were computed using bridge sampling (Gronau et al. 2017), comparing models with and without the factor of interest (i.e., in the baseline model the coefficient of interest is absent, whereas it is present in the full model).

##### 2.5.2.1 Pruning the Model

If the model with our maximal random effects structure did not converge, we planned to prune the random effects structure such that random slopes for less theoretically central effects were removed first. We first pruned random slopes for interactions, followed by random slopes for labs, and then random slopes for participants. Random intercepts were pruned last, again removing random intercepts for labs before random intercepts for participants. Together, this resulted in the following order for removing random effects:

1. Subject‐level random slope for the interaction of trial_type and trial_num
2. Lab‐level slope trial_type
3. Lab‐level slope test_order
4. Subject‐level slope trial_num
5. Subject‐level slope trial_type
6. Lab intercept
7. Subject intercept

##### 2.5.2.2 Hypothesis Tests

In Table 6 below we provide the model terms, their connection with specific hypotheses, and their expectation (positive, negative, null). Note that indeed some effects are expected to be null, and their being null is informative to the literature, hence Bayes factors are required to test these models. Note that not all models terms are included in the table because they do not correspond to any specific hypotheses such as some interaction and random effects.

**TABLE 6:** Hypotheses, statistics and expected direction of effects.

Hypothesis	Model term	D
Hypothesis 1a: Rule learning is evidenced by a novelty preference for inconsistent patterns at test (with “same” as baseline).	trial_type	+
Hypothesis 1b: After controlling for age, linguistic background, and experimental paradigm, there is little residual heterogeneity between labs (as assessed by Cochran's *Q*).	Random variances across lab	0
Hypothesis 2a: Rule learning is robust across age and increases with age.	age:trial_type	+
Hypothesis 2b: Looking times decrease with test trial; this effect will be stronger for older infants than younger infants.	trial_num:age	+
Hypothesis 2c: The relationship between multilingualism and rule learning will be assessed*.	multilingual_exposure: trial_type	+/0/‐
Hypothesis 2d: Rule learning is robust across variation in experimental paradigms and is if anything smaller in non‐HPP procedures (i.e., Headturn Preference Procedure will serve as baseline).	experimental_method: trial_type	‐/0
Hypothesis 3a: There is expected to be an overall preference, i.e., longer looking times, for direct repetition items in the test phase, i.e., ABB items.	repetition	+
Hypothesis 3b: There will be a larger effect of learning overall in the participants trained on ABB than in the participants trained on ABA (with ABA as baseline).	familiarization: trial_type	+

*Note*: Hypotheses are those listed in the introductory sections; model term has the R code specification of the model coefficient testing the particular hypothesis; D denotes the expected sign of the coefficient (+ or ‐ or 0; the latter being for expected null effects). *Note that this answers an exploratory question rather than tests a confirmatory hypothesis.

#### 2.5.3 Additional Hypotheses

A number of additional hypotheses were planned to be tested if deemed useful given the main results and if sufficient data permitted. Only the first of these was ultimately tested (for completeness, the others are included in the Supplementary Materials):

- Do we replicate the original Marcus et al. (1999) study when considering only the data from populations (i.e., 7‐month‐old English learners) and methods (i.e., HPP) that most closely match the original design? This analysis follows the main analysis, and uses Bayes factors to determine the degree of support for this replication, including whether there is sufficient data to perform the comparison. The key model term is trial_type.

#### 2.5.4 Robustness Analyses

In order to check the robustness of our results, we ran some checks. In particular, we did this in the form of a multiverse analysis (Steegen et al. 2016). A large advantage of pre‐registering a study prior to performing it and running the analyses is that we were forced to be as specific as possible in laying out the choices that were made in pre‐processing the data, determining exclusion criteria, and the analyses that were performed. Many of these choices could be well justified based on previous research. For some choices, however, it was simply unknown what their effects would be. In all of these cases it is interesting to find out whether our main results would be robust to making different choices. Hence, here we re‐ran the main model when different choices were made. Here we only list the robustness analyses that were eventually performed and reported in Results (the complete list is in the Supplemental Materials):

1. **Minimal valid looking time per trial**: the minimal valid looking time for the main analysis is set at 1 s as that is the minimum time needed to disambiguate whether the currently presented trial follows an ABA versus an ABB pattern. In many looking time studies, a 2 s minimum looking time is used, hence in this robustness analysis we included only trials with a 2 s minimal looking time.
2. **Minimum number of trials**: in the main model we include infants with as few as 2 out of 12 valid trials, assuming these trials are of different type; it could be argued that a larger minimum is required to reliably assess any novelty/familiarity effect; we include thresholds of 3, 4, 6, 9, 12 respectively to test the robustness of the results (in all cases also assuming a minimum of 1 same/1 different trial).
3. **Test‐trials within a block**: in the main model we included infants with a minimum of 2 completed test trials, one “same” and one “different”; as a result, these test‐trials can be very far apart (spanning several invalid trials) which could potentially lessen the rule learning effect; here, instead, we only include infants if they had a pair (one “same,” one “different”) of valid test‐trials within a block of 4 trials.
4. **Trial spill over effects**: In the main model we include trials that are collected after a trial that went missing due to trial‐level errors; here we exclude all trials that follow an initial missing trial due to infant factors as some of the effects may spill over from the missing trial.

Together, these robustness checks can help determine the generalizability of our results in the face of different analytical choices. In other words, they can strengthen the confidence in our results given consistent results across these choices. In the case of inconsistent results, these checks can help determine causes of variability in infant looking time research.

## 3 Results

The data collected in this study is available through a github repository which also includes the code used for data pre‐processing and analyses: MB3‐analysis‐public (https://github.com/manybabies/mb3-analysis-public/).

Before analyses were performed, data was blinded by scrambling the condition variable such that the analysis team was blind to the results while writing and testing the code required to perform the analyses. Data were unblinded in May 2024 after a period of code writing and data validation.

**FIGURE 1:**
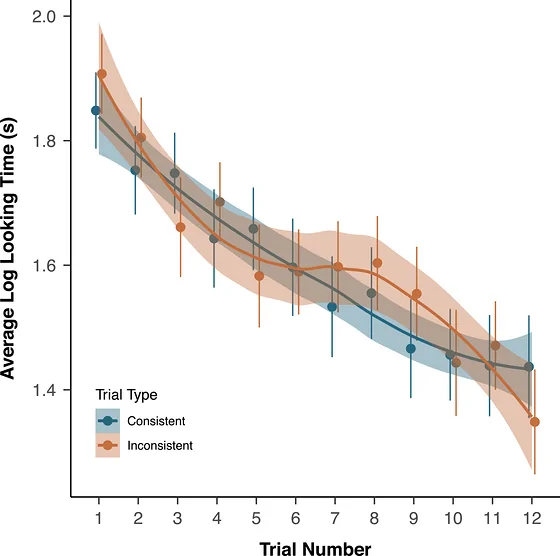
Log looking times for consistent and inconsistent test trials. *Note*. Lines represent a LOESS fit with 95% confidence bands. Error bars represent 95% confidence intervals.

The main variable of interest is the looking time during consistent versus inconsistent trials at test. Table 7 provides the raw average looking times by trial and for consistent and inconsistent trials separately. As analyses are performed on the log‐transformed looking times, Figure 1 depicts the log looking times for test trials 1 through 12, for consistent and inconsistent trials separately.

**TABLE 7:** Average looking times (in seconds) by trial number and trial type.

Trial	Consistent	Inconsistent
1	7.27 (3.70)	7.71 (3.78)
2	6.85 (3.87)	7.00 (3.83)
3	6.85 (4.07)	6.41 (3.90)
4	6.28 (3.67)	6.57 (3.95)
5	6.31 (3.88)	6.11 (3.98)
6	6.04 (3.80)	5.83 (3.49)
7	5.74 (3.83)	6.10 (3.90)
8	5.84 (3.72)	5.90 (3.62)
9	5.35 (3.63)	5.75 (3.66)
10	5.24 (3.56)	5.23 (3.46)
11	5.23 (3.60)	5.26 (3.44)
12	5.30 (3.73)	4.94 (3.64)

*Note*: Average looking times and standard deviations in parentheses.

### 3.1 Hypothesis Testing

To test the main three hypotheses, the model specified in the methods sections was fitted (see section Statistical modeling). All numerical variables were scaled (age, trial number and multilingual exposure) and effect coding was used for binary variables—making for more stable estimation and easier interpretation of the intercept. Relative to the registered report there was one change to the model. Rather than using dummy coding (with HPP as the baseline), we now use deviation coding for the methods variable (Barr 2021), as this allows us to interpret the trial type predictor as the overall novelty preference averaging across the three experimental paradigms (akin to a main effect in an ANOVA approach), rather than the effect of trial type for a specific experimental paradigm (i.e., HPP). Note that we also fitted the model from the registered report (i.e. using dummy coding) leading to identical conclusions. The model was initially fitted with a full random effects structure. The random effects structure in the final model contains random effects for participants and labs; all other random effects were pruned (in the order specified in the Methods section).

The parameter estimates for the resulting model are presented in Table 8 including *p*‐values, Bayes factors and confidence intervals for the model coefficients. Bayes factors were added to be able to better interpret coefficient values with non‐significant *p*‐values.

**TABLE 8:** Parameter estimates and hypotheses tests.

Factor	coeff	low	up	se	stat	df	*p*	BF10
Intercept	1.59	1.50	1.68	0.04	36.43	28	0.000	1.22e+20
FamRule	−0.03	−0.16	0.10	0.07	−0.49	1517	0.626	0.177
Type	0.00	−0.03	0.02	0.01	−0.34	6206	0.738	0.034
Age	−0.02	−0.05	0.02	0.02	−0.98	622	0.328	0.061
Method (CF)	0.10	−0.12	0.32	0.11	0.95	35	0.348	0.44
Method (ET)	−0.17	−0.36	0.01	0.09	−1.89	68	0.064	1.048
Lang Exp	0.02	−0.01	0.05	0.02	1.20	630	0.231	0.079
Trial	−0.14	−0.15	−0.13	0.01	−22.34	6271	0.000	1.26e+102
Rep	−0.10	−0.23	0.04	0.07	−1.40	6225	0.162	0.473
FamRule*Type	−0.06	−0.19	0.08	0.07	−0.86	6225	0.389	0.255
Type*Age	0.01	−0.02	0.03	0.01	0.54	6207	0.592	0.033
Type*Method (CF)	−0.03	−0.10	0.04	0.04	−0.83	6204	0.406	0.11
Type*Method (ET)	−0.01	−0.08	0.05	0.03	−0.44	6208	0.660	0.085
Type*Lang Exp	0.01	−0.02	0.03	0.01	0.50	6204	0.614	0.033
Type*Trial	0.00	−0.03	0.02	0.01	−0.09	6230	0.926	0.03
Age*Trial	−0.02	−0.03	0.00	0.01	−2.74	6297	0.006	0.632
Type*Rep	−0.15	−0.67	0.36	0.26	−0.58	1610	0.565	0.784

The complete model with fixed and random effects fits the data well and there is little residual heterogeneity; a more formal test of the latter is reported below.

The first research question concerns the rule‐learning effect, operationalized as a novelty effect for inconsistent patterns at test (hypothesis 1a). Table 8 indicates that this effect (trial_type) is non‐significant. There is no evidence for rule‐learning. The Bayes factor corresponding to the effect of trial_type indicates that in fact there is strong evidence for the contrary, that is, evidence for a null effect of trial_type; the corresponding Bayes factor, $BF_{01}\left(trial\_type\right)=29.72$ (being the inverse of the Bayes factor for this effect in the Table 8 which is $BF_{10}$).

The second research question asks whether rule‐learning is moderated by age, language background and experimental paradigm (HPP, CF, ET). None of these moderators significantly affect rule‐learning as can be seen from both *p*‐values for the corresponding coefficients in the Table 8, that is, the coefficients of the interaction between trial_type and the moderating factors of age, multilingual exposure and the experimental paradigm (cf and eyetracker separately, with HPP being the baseline). The Bayes factors for each of these coefficients indicate that not only are the coefficients non‐significant, but to the contrary, there is strong evidence that they are null effects. The Bayes factors in favor of the null hypothesis are, respectively: $BF_{01}\left(trial\_type:age\right)=30.41$, $BF_{01}\left(trial\_type:multilingual\_exposure\right)=30.24$, and $BF_{01}\left(trial\_type:method\_cf\right)=9.1$ & $BF_{01}\left(trial\_type:method\_et\right)=11.82$.

The third research question asks whether looking times and rule‐learning are impacted by the familiarization rule, that is, whether the familiarization rule with a direct repetition impacts looking times and rule‐learning. Results indicate that this is not the case: the coefficients for familiarization rule and repetition and their interaction effects with trial type are all non‐significant. The corresponding Bayes factors indicate evidence for null effects.

As auxiliary hypotheses, we included age, trial number at test, and their interaction as moderators of looking times irrespective of trial type. As expected, looking times significantly decrease with trial number as a result of habituation, whereas age does not significantly affect looking times; the interaction between age and trial number does significantly impact looking times. As can be seen in the Table 8, the Bayes factor for the trial number effect shows extremely strong evidence with the log BF>200.

**FIGURE 2:**
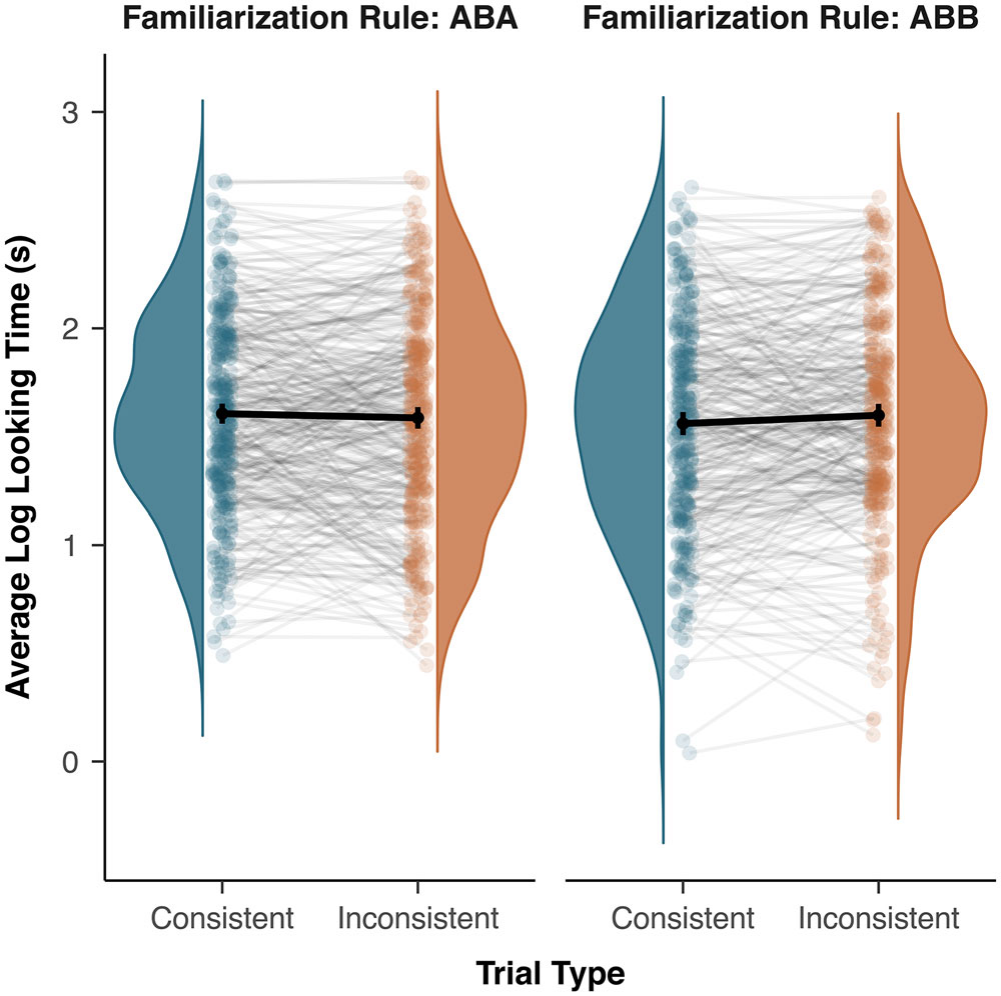
Effect of Trial_type (consistent vs. inconsistent with the familiarization rule) split by familiarization rule (ABA and ABB). *Note*. The *y*‐axis depicts average log looking times. Points represent individual participants' average log looking time, with lines connecting looking times by a given participant across trial types, in order to visualize within‐participant effects. Violin plots visualize the distribution of individual participants' average log looking times. Error bars represent 95% CIs.

The rule‐learning effect is further depicted in Figure 2 by plotting the looking times for consistent and inconsistent items at test, split by familiarization rule. Note that there are error bars around the means representing confidence intervals.

**FIGURE 3:**
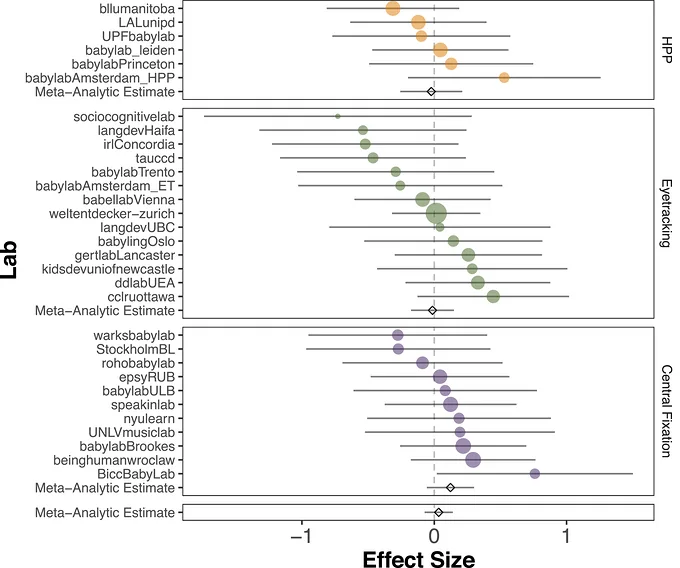
Forest plot of standardized effect sizes per lab. *Note*. Error bars show 95% confidence intervals. Labs are grouped by method. Points are scaled by inverse variance and colored by experimental paradigm. In each panel, the diamond and associated interval represent the meta‐analytic estimate from the method‐moderated model and its 95% confidence interval. The bottom panel shows the global meta‐analytic estimate from the unmoderated model.

After finding evidence against the presence of rule‐learning in the current data set, next we checked the effect sizes per lab to study potential differences in the findings between labs. Figure 3 depicts the effect sizes (Cohen's *d*) per lab of the rule‐learning effect (i.e., the effect of trial type) including their confidence intervals. The effect sizes are clustered by testing method. Figure 3 also depicts the meta‐analytic effect sizes by method and overall.

Figure 3 indicates that in 30 out of 31 of the samples, the effect size was not significantly different from zero. The exception is the BICC Babylab with a valid sample size of 15. The meta‐analytic combined effect sizes for each of the methods as well as the overall meta‐analytic effect size are not significantly different from zero.

#### 3.1.1 Residual Heterogeneity

The next analysis that we performed corresponds to Hypothesis 1b, using a meta‐analytic modeling approach. We compute Cochran's *Q* in a moderated model including age, linguistic background, and experimental paradigm to test whether there is any residual heterogeneity left after controlling for these factors.

The Cochran's *Q* in the meta‐analytic model including the moderators of average age (centered and scaled), average multilingual exposure (centered and scaled), and experimental paradigm (dummy coded, with CF as the reference level) was *Q = 24.34*, *p = 0.56*. This means there is no significant residual heterogeneity left after including these moderators.

#### 3.1.2 Multi‐Lingual Exposure and Experimental Paradigm Effects

**FIGURE 4:**
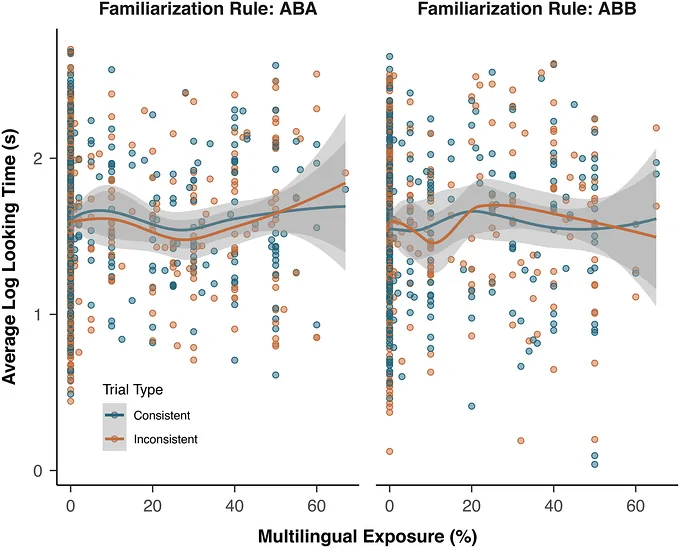
Rule learning effect by percentage exposure to other languages than the primary language.

**FIGURE 5:**
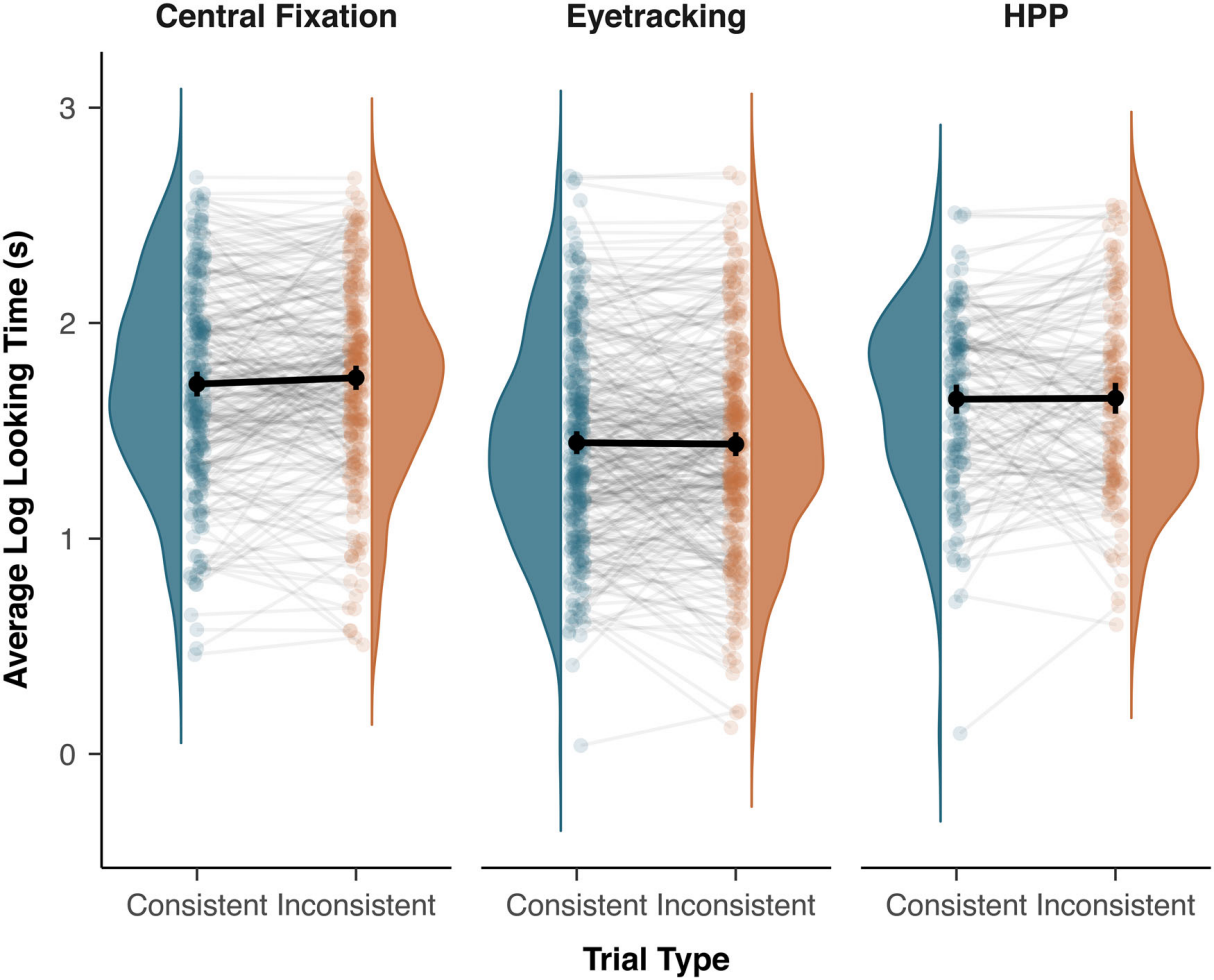
Effect of Trial_type by experimental paradigm (central fixation, eyetracking, HPP = Headturn Preference Procedure). *Note*. Points represent individual participants' average log looking time, with lines connecting looking times by a given participant across trial types. Violin plots visualize the distribution of individual participants' average log looking times. Error bars represent 95% CIs.

Figure 4 corresponds to Hypothesis 2c, depicting the relationship between rule learning and multi‐lingual exposure. Figure 5 corresponds to the question in Hypothesis 2d, studying the effect of experimental paradigm on the rule learning effect.

None of these moderators significantly affect rule‐learning as can be seen from both *p*‐values for the corresponding coefficients in the Table 8, that is, the coefficients of the interaction between trial_type and the moderating factors of age, multilingual exposure (Figure 4, corresponding to Hypothesis 2c) and the experimental paradigm (cf and eyetracker separately, with HPP being the baseline).

### 3.2 Additional, Robustness, and Exploratory Analyses

In the registered report we proposed a number of additional analyses in case the data were sufficiently large to warrant testing them. The rationales for these additional hypotheses (except one) were reliant on finding a rule‐learning effect. Given the lack of such effect, these analyses are no longer well‐motivated. We therefore only include results from the first additional hypothesis concerning whether in our sample we can replicate the original findings from Marcus et al. Specifically, in this analysis we limit the data to the age group and the paradigm from the original study by Marcus et al., that is, 7‐month‐olds with English as their primary language, performing in the HPP. Results indicate no evidence for rule‐learning in this subset of the data (*p = 0.51*, *n = 19*, cf. *n = 16* in Experiment~2 of Marcus et al.); the full model results are in the Supplemental Materials.

In the registered report we made a number of choices pertaining to data selection. In the preregistered robustness analyses we investigate the consequences of these choices on the effects of interest. We do this in each case by constructing a new dataset (e.g., by including only participants with 4 or more valid test trials—rather than the criterion of a minimum of 2 trials that was used in the main analysis) and running the main model on this dataset. Each model is started with the same random effects structure as was found in the main model (that is, the main model that remained after pruning) and further pruning was done in the preregistered order if necessary.

**FIGURE 6:**
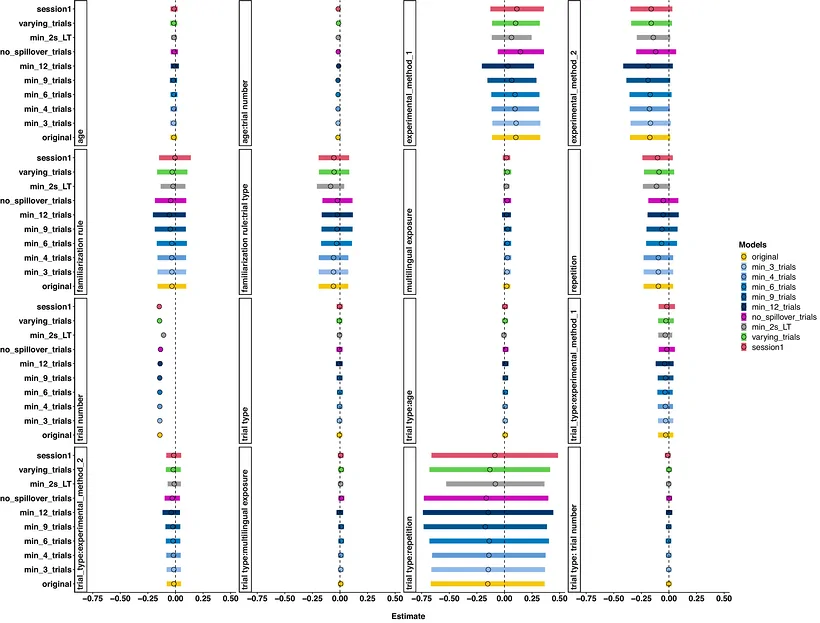
Robustness analysis results (see text for details).

Figure 6 depicts the coefficients and their confidence interval for each data selection. The most relevant coefficient is the effect of trial_type and the graph clearly shows that the confidence intervals for each of the estimates includes zero for all data selections that the models were fitted to.

Some of the pre‐registered robustness analyses were not performed for various reasons resulting in insufficient/missing data, see Supplementary Materials for details.

During data collection, a technical error at one lab resulted in familiarization times being twice as long as intended (4 min instead of 2); these data were left out of the main analysis. However, this gave us the unique opportunity to exploratorily check whether longer familiarization time would result in rule learning. The fitted model shows a non‐significant effect of trial_type on looking_time (β *= ‐0.05*, *p = 0.3*) and hence there is no evidence of rule learning in this sample. The second exploratory analysis concerns the question whether the rule‐learning effect may be short‐lived and only present in the first few trials. Here analysis is limited to the first four trials, and only includes infants that have at least 2 valid trials within those first four trials (as was the case in the main analysis). The fitted model shows a non‐significant effect of trial_type on looking_time (β *= 0.46*, *p = 0.65*). The full model results for both models are presented in Supplemental Materials.

## 4 Discussion

### 4.1 Summary and Main Findings

This large‐scale cross‐linguistic study (30 labs; 650 analyzed participants) aimed to provide a conceptual replication of the seminal work by Marcus et al. (1999), which proposed that infants possess rule‐learning abilities. The Marcus et al. (1999) findings, along with numerous subsequent studies (e.g., the studies mentioned in the meta‐analysis by Rabagliati et al. 2019), have been central to theories of language acquisition (Geambaşu et al. 2023). This includes debates about the type of learning mechanisms that support language development, whether learning mechanisms are domain‐specific or domain‐general, and the nature of the underlying representations that infants use to learn new patterns (cf. Seidenberg 1997). Given the theoretical importance of rule‐learning abilities, we developed a phonologically balanced and broadly discriminable set of stimuli in order to provide a conceptual replication of Marcus et al.'s findings in infants aged 5–12 months from diverse language backgrounds (33 different primary languages). Contrary to Marcus et al.'s findings, our results provide strong evidence in favor of the null hypothesis, showing no significant rule‐learning effect in the overall data or at the individual lab level except in one lab (RQ1). The robustness analyses, which aimed to assess the impact of methodological and analytical decisions, revealed that this absence of a rule‐learning effect persisted across various analytic decisions, including analyzing only the initial trials, varying the minimum looking time per trial, and the number of valid trials per infant required for inclusion. Further analyses revealed that we can be extremely confident that the absence of the rule‐learning effect was not influenced by age, experimental paradigm, or linguistic background (RQ2). While our study did not directly address whether infants might succeed with longer familiarization times, data from one lab involved in the current study suggest that even with a longer, 4‐min familiarization, no rule learning effect was observed. Finally, the null finding, supported by Bayesian analyses, was unaffected by whether the infant was familiarized with ABA or ABB patterns; therefore, direct repetition did not facilitate rule‐learning in our study (RQ3).

These strong null findings prompt a reconsideration of what capabilities infants bring to the task of language learning. Before we turn to a discussion of some of the wider implications of our findings, we first consider the possible influence of our stimuli on the findings, since this aspect of our study diverged in several ways from the original study and could thus arguably have played a role in the difference between our findings and the original findings from Marcus et al. (1999).

### 4.2 Could the Nature of the Stimuli Explain the Null Results?

There are five ways in which our speech stimuli differed from the original study by Marcus et al. (1999). First, where Marcus et al. (1999) used synthesized speech syllables, our study used naturally spoken syllables. We anticipated that more natural speech stimuli would facilitate, rather than impede learning, simply because they are more language‐like (cf. Rabagliati et al. 2019). Second, the original stimuli featured sounds that are specific to English, such as the affricate 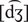 and vowels 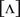 and [aı]. In contrast, our stimuli only featured sounds that are common in many languages, such as the voiceless bilabial plosive [p] and the vowel [a]. This deviation was necessary as the current study included and tested infants with diverse language backgrounds, which ensured a greater generalizability of our results. Third, unlike the original study, which did not control for durational and intonational differences in the syllables used in the speech stimuli, the syllables in the current study were edited to minimize potential confounds. Prosodic features such as mean amplitude, duration, and f0 were carefully controlled, ensuring consistency across all stimuli. One could argue that the presence of intonation and duration differences may have facilitated the discovery of rules in the original study, and that by removing these features infants were no longer able to detect rules. However, this is unlikely because follow‐up studies that did report a rule learning effect also used stimuli that were controlled for mean amplitude, duration, and f0 (e.g., Kovács and Endress 2014; Pons and Toro 2010). Fourth, unlike the original stimuli, we paid particular attention to the discriminability of the As and Bs in our stimulus set, by making sure that the pattern was present in both the consonants and the vowels. As described in the Methods section, A and B syllables never contained similar vowels or consonants, which could, especially in the case of similar vowels (Thiessen 2012) hinder the discovery of ABB or ABA patterns. Fifth, one could argue that our stimuli were not “meaningful” enough compared to the original stimuli. Rabagliati et al. (2019) showed in a meta‐analysis that more meaningful stimuli are advantageous for rule learning in infants. This included speech, but also other types of stimuli of social or perceptual experiences, such as pictures of dogs. However, there is no convincing reason to assume that our stimuli were more or less “meaningful” than the stimuli used by Marcus et al. (1999).

In addition, it should be emphasized that a symbolic rule‐based interpretation — suggesting the involvement of innate learning abilities — is an abstract proposition that should accommodate variability in tokens, that is, the rule ABA applies to any discriminable As and Bs, and should not be highly sensitive to small changes in stimulus characteristics. As mentioned above and described in our Methods section, we have paid particular attention to this discriminability. Taken together, this suggests that the null results in the current study are unlikey to be solely attributed to stimuli differences. It cannot be ruled out that some unidentified property of our stimuli or design may have impeded the infants' true rule learning capability; however, rule learning as a robust effect should have emerged within the breadth of measures and ages in our large sample. Finally, a study we mentioned in the introduction has been published since the in‐principle acceptance of this registered report, showing that 7‐month‐old Dutch infants did not demonstrate a rule learning effect (Geambaşu et al. 2023) using the same stimuli as Marcus et al. (1999). This further strengthens the argument that the null findings are unlikey to be attributable to the stimuli alone, as even studies employing the same stimuli fail to report a rule learning effect.

### 4.3 Theoretical Implications

Given the theoretical importance of the Marcus et al. (1999) study and the many studies that followed, the failure to replicate the results of the original study raises a few fundamental questions. First of all, it is important to address explicitly how our null findings intersect with the larger debate in the literature about rule learning. Marcus et al. 1999 argued that their findings supported the idea of symbolic rule learning, suggesting that infants are capable of learning abstract, grammatical rules that go beyond mere pattern recognition. They claimed that simple neural networks, which rely on statistical learning rather than explicit rules, would fail to replicate their results. In response to this claim, several successful replications of the findings using artificial neural networks were published (e.g., Altmann and Dienes 1999; Seidenberg and Elman 1999; Sirois et al. 2000), thereby challenging the symbolic interpretation. However, 25 years on from this debate, the symbolic versus statistical learning dispute remains unresolved (Alhama and Zuidema 2019). This is in part because findings are difficult to replicate or go in the opposite direction (Gerken 2006). However, a further complication in the literature is that we are potentially confusing findings and interpretation when we discuss such tasks as evidence of rule learning (symbolic) or statistical learning, although the latter benefits from parsimony in the absence of a litmus test for either. The absence of evidence for rule learning in our study challenges the claim that infants learn abstract, grammatical rules, but it does not resolve the ongoing debate about whether rule learning can be explained by neural network models that do not rely on symbolic representations.

In fact, the evidence we report here points against a rule learning effect in contrast to some other findings in the literature. If our finding is capturing the true (null) effect, what does this suggest about the wider literature that does report such effects? Some of these studies may have reported inflated effect sizes, possibly due to factors such as small sample sizes, limited statistical power, or random noise. However, some studies may be detecting effects that reflect infants' (or adults') sensitivity to features of the stimuli other than the intended “rule,” such as repetition or surface‐level patterns, rather than abstract rule learning per se (though note that we did not find evidence for a repetition effect in the current study despite the design including a familiarization rule with a direct repetition). Therefore, findings previously reported in the literature may still provide crucial insights into cognitive processes involved in early learning mechanisms even if our null result represents the true underlying rule‐learning capability of infants. However, for rule learning to be considered a fundamental aspect of early infant learning, it must be consistently and robustly observed across studies.

### 4.4 Implications for Large‐Scale Replication Work

Large‐scale replication studies, like the one presented here, are essential to building a more comprehensive understanding of early cognitive abilities. It is worth commenting briefly on the implications of this work in light of similar recent efforts from the ManyBabies framework, particularly given the significant resources these projects command. While the original ManyBabies study successfully replicated a preference for IDS (Consortium 2020), in three recent efforts [the current study, ManyBabies 2, and ManyBabies 4; Schuwerk et al. (2025); Lucca et al. (2025)], the results have challenged cornerstone findings of seminal studies in the developmental literature. These seminal studies have “rippled” through the field, shaping subsequent research directions and driving new insights. The fact that a cornerstone finding is called into question does not necessarily invalidate the studies that have built upon it. The body of findings reported in subsequent studies still holds significant value. However, it does prompt us to reconsider and refine our understanding of the literature as a whole.

One important question is the relative value of large‐scale data collection efforts compared to meta‐analyses that leverage existing smaller studies in the literature for similar goals. Meta‐analyses and large‐scale collaborations both attempt to establish more clearly the true effect size of a phenomenon and examine moderators of interest using large datasets. While large‐scale collaborations work to reduce variability across a large sample and systematically vary a small number of factors of interest, meta‐analyses work with the existing variability in the literature to extract meaningful insights. While both methods have a similar goal, their results do not always agree. Recent work comparing meta‐analyses to multi‐lab replications found that meta‐analytic effect sizes tend to be systematically larger than replication effect sizes (Kvarven et al. 2020). Consistent with this result, the current study found an effect size indistinguishable from zero, while an existing meta‐analysis of rule learning found a robust rule learning effect for “meaningful” stimuli (Rabagliati et al. 2019). These findings stand in contrast to a recent study which found that the meta‐analytic effect size of IDS preference was similar to the effect size estimated in the multi‐lab collaboration ManyBabies 1 (Zettersten et al. 2024). How can we explain discrepancies between meta‐analytic results and multi‐lab replications, both generally and in the specific case of rule learning? Future work could expand the existing meta‐analysis of rule learning (Rabagliati et al. 2019) by including findings published since the meta‐analytic effect was first reported and systematically coding additional study features (e.g., related to stimulus characteristics discussed above), to examine whether moderating factors can explain differences in effect size estimates derived from the current study and from meta‐analytic evidence (Lewis et al. 2022). A more in‐depth analysis of potential publication bias — which impacts meta‐analytic studies, but not multi‐lab studies — could provide additional insights in this field of study.

As both replication and meta‐analytic efforts continue to grow, one important consideration is the potential for cohort effects to complicate the interpretation of broad disciplinary findings (this is another reason why examining publication year would be valuable). The early seminal studies and the current multi‐lab replication attempt are spanned by two decades or even more. The social and linguistic environments in which infants grow up have undoubtedly changed across the original and the replication studies, which might be reflected in how infants responded in the earlier versus in the recent lab‐based studies (e.g., changes in infant attention or changes in sampling biases over time). Besides refining the methodology of the earlier studies and increasing the diversity of studied populations, future replication work could also consider potential environmental/experiential factors, and directly compare them, to examine their impact on effects across studies.

Taking a step back, there are two implications of our findings. First, it is an important reminder of the value of null results in the scientific process (The importance of no evidence, (Editorial 2019). Ultimately, this study highlights the need for replication in developmental psychology research, particularly when it comes to cornerstone findings that shaped the field. Null findings not only challenge existing assumptions, but also guide future researchers toward refining theories and exploring alternative explanations, thereby advancing the field. Second, they force us to consider the relationship between the theory being tested and the methods used to investigate it. For example, the possibility that infants show rule learning effects via other measures (e.g., pupillometry or fNIRS) is being explored in two MB3 spin‐off projects (MB3P, MB3N). To what extent are variations in outcomes evidence of theoretically interesting variations in infants' abilities in different contexts, and to what extent are they evidence of a lack of “robustness” of an effect that could bring the theory itself into question? These are concerns that we as a discipline need to consider head on.

## 5 Conclusion

This large‐scale cross‐linguistic study challenges the foundational findings of Marcus et al. (1999), providing robust null results and showing clear evidence against successful rule learning by infants across diverse linguistic backgrounds and different ages. These findings were consistent across methodological variations, and align with a recent close replication failure (Geambaşu et al. 2023), and with other recent failed multi‐site replication attempts of other seminal infant cognition phenomena (e.g., Lucca et al. 2025; Schuwerk et al. 2025). This study's results suggest at a minimum that infants' ability to demonstrate rule‐learning capabilities are context‐dependent rather than robust across testing frameworks. Our strong null results highlight the need to reconsider the developmental trajectory of rule learning, suggesting alternative theoretical frameworks may be necessary to capture the infant's core language learning process, or that infants' rule learning behaviors are dependent on specific characteristics of stimuli which are still to be determined. Making further progress will require moving beyond the debates about algebraic rule learning in which we have been mired for decades, and towards a deeper investigation of the underlying processes and constraints that shape early learning.

## Funding

Benedetta Colavolpe was supported by “SYNPHONIA” (nr. PNRR‐MAD 2022‐12376739) project founded by the European Union Next Generation EU (NRRP M6C2, Investment 2.1 “Enhancement and strengthening of biomedical research in the NHS” (A.R.F., P.T.), ERC Consolidator Grant “BabyRhythm” no. 773202 (J.G.).; Kateřina Chládková was supported by CSF 21‐09797S; Nicolás Alessandroni was supported by Concordia Horizon Postdoctoral Fellowship, FRQSC Postdoctoral Fellowship (#333109); Monika Kučerová was supported by Czech Science Foundation grant no. 21‐09797S Czech Academy of Sciences project no. LQ300252401; Martina Turconi was supported by ERC Consolidator Grant “BabyRhythm 773202”, the ECOS‐ Sud action nr. C20S02, a FARE grant nr. R204MPRHKE from the Italian Ministry for Universities and Research, the Italian Ministry for Universities and Research PRIN grant nr. 2022WX3FM5 and a PNRR‐MAD‐2022–12376739 Grant (“SYNPHONIA”, European Union, Next Generation EU – PNRR M6C2 ‐ Investimento 2.1) awarded to Judit Gervain.; ZEYNEP AYDIN was supported by ERC Consolidator Grant “BabyRhythm 773202”, the ECOS‐ Sud action nr. C20S02, a FARE grant nr. R204MPRHKE from the Italian Ministry for Universities and Research, the Italian Ministry for Universities and Research PRIN grant nr. 2022WX3FM5 and a PNRR‐MAD‐2022.12376739 Grant (“SYNPHONIA”, European Union, Next Generation EU – PNRR M6C2 ‐ Investimento 2.1) awarded to Judit Gervain. The Italian National Graduate Program in Theoretical and Applied Neuroscience PhD fellowship DM.352 supported by MUR (PNRR program) to Zeynep Aydin and Judit Gervain.; Judit Gervain was supported by ERC‐2017‐COG ‐ 773202 BabyRhythm; Gert Westermann was supported by ESRC ES/S007113/1; Wiktoria Jędryczka, Michal Misiak, and Piotr Sorokowski were supported by IDN Being Human funds; Sagi Jaffe‐Dax was supported by ISF 336/22; Hermann Bulf was supported by Italian Ministero dell'Istruzione dell'Universitá e della Ricerca, PRIN 2022 No. 2022‐NAZ‐0410; Jeanne L. Shinskey was supported by Nuffield EDO/43951; Martina S. Zaharieva was supported by Research Priority Area Yield of the Faculty of Social and Behavioural Sciences, University of Amsterdam; Alexis K. Black was supported by SSHRC IDG 430‐2020‐00430; SSHRC IG 435‐2024‐0782; Melanie Soderstrom was supported by SSHRC Partnership Development Grant 890‐2020‐0059; Iris‐Corinna Schwarz was supported by Stockholm University Black Box Dnr SU FV‐3299‐22; Elísabet Eir Cortes and Ellen Marklund were supported by Stockholm University Black box Dnr SU FV‐3299‐22.

## Conflicts of Interest

The authors declare no conflicts of interest.

## Supporting information

Data S1

## Data Availability

The data that support the findings of this study are openly available in mb3‐analysis at https://github.com/manybabies/mb3‐analysis‐public/.

## Endnotes

1 Detailed instructions for participating labs can be found in the ManyBabies 3 Lab Manual on the MB3‐OSF repository (https://osf.io/kqu9v/overview).

2 For context, *d* = 0.25 is the average effect size from a meta‐analysis of published rule learning studies (Rabagliati et al. 2019), while *d* = 0.3 is the average effect size across all published developmental experiments. *d* = 0.2 is generally considered a “small” effect size, and thus *d* = 0.1 is quite small.
