# Supplementary material for: ManyBabies 3: A Multi‐Lab Study of Infant Algebraic Rule Learning
Source: Dev Sci. 2026 Sep 15;29(6):e70259. doi: 10.1111/desc.70259 (PMC13578860; doi:10.1111/desc.70259)
Supplement: Supplementary file 1 [file DESC-29-e70259-s001.pdf]

ManyBabies 3: A Multi-Lab Study of Infant Algebraic Rule Learning: Supplemental Material

This report contains Supplemental Material for the ManyBabies 3 study on rule learning.

Additional hypotheses

Here we include the complete list of additional hypotheses as specified in the Stage 1 report. Only the first of these was eventually tested, as the others are not informative in light of the null results found in the main analysis.

1. Do we replicate the original Marcus et al. (1999) study when considering only the data from populations (i.e., 7-month-old English learners) and methods (i.e., HPP) that most closely match the original design? This analysis follows the main analysis, and uses Bayes factors to determine the degree of support for this replication, including whether there is sufficient data to perform the comparison. The key model term is trial_type.

Modeling results for this analysis are presented in Table 1. As can be seen, there is no evidence for a rule learning effect in this subsample, p = 0.51 for the trial_type effect.

2. Rule learning is robust and does not vary across primary languages. Related to the universality of rule learning we planned to test whether rule learning varies with infants' primary language. As the number of primary languages was predicted to be large and collinear with laboratories this would only be explored after fitting the main model. This was planned to be tested by including the primary_language:trial_type fixed effect into the model.

3. There is an interaction effect between age and non-adjacent repetition (i.e. ABA), with older infants showing more learning from ABA training stimuli than younger infants. This hypothesis can be tested by including familiarization:trial_type:age into the model. This being a 3-way interaction in an already complex model, we planned to only add this after fitting the main model.

4. The relationship between multilingualism and non-adjacent repetition learning was planned to be explored. Either negative, positive or null result would inform the debate about

Table 1

Parameter Estimates and P-values for the 'Marcus et al' Dataset.

factor	coeff	se	stat	df	p
Intercept	1.60	0.05	33.39	19	0.000
FamRule	-0.04	0.14	-0.29	49	0.775
Type	0.04	0.07	0.65	204	0.513
Lang	0.10	0.05	1.87	20	0.076
Trial	-0.15	0.03	-4.64	208	0.000
Rep	-0.34	0.23	-1.46	204	0.144
FamRule*Type	-0.39	0.23	-1.71	204	0.089
Type*Lang	-0.05	0.07	-0.69	204	0.490
Type*Trial	0.05	0.07	0.81	207	0.418
Type*Rep	-0.29	0.57	-0.52	47	0.608

potential cognitive benefits for multilingual infants. This can be tested by adding familiarization:trial_type:multilingual_exposure into the model. Similar to the previous hypothesis 3, this involves a 3-way interaction and hence we would only add this after fitting the main model.

Robustness analyses

The complete list of robustness analyses as specified in the Stage 1 report is included here, detailing below each whether it was run or not, and if not, why not.

1. **Minimal valid looking time per trial**: the minimal valid looking time for the main analysis is set at 1 s as that is the minimum time needed to disambiguate whether the currently presented trial follows an ABA versus an ABB pattern. In many looking time studies a 2 s minimum looking time is used, hence in this robustness analysis we included only trials

with a 2 s minimal looking time. This factor was included in the robustness analyses, see the main report for the results.

2. **Minimum number of trials**: in the main model we include infants with as few as 2 out of 12 valid trials, assuming these trials are of different type; it could be argued that a larger minimum is required to reliably assess any novelty/familiarity effect; we include thresholds of 3, 4, 6, 9, 12 respectively to test the robustness of the results (in all cases also assuming a minimum of 1 same/1 different trial). This factor was included in the robustness analyses, see the main report for the results.

3. **Test-trials within a block**: in the main model we included infants with a minimum of 2 completed test trials, one 'same' and one 'different'; as a result, these test-trials can be very far apart (spanning several invalid trials) which could potentially lessen the rule learning effect; here, instead, we only include infants if they had a pair (one 'same', one 'different') of valid test-trials within a block of 4 trials. This factor was included in the robustness analyses, see the main report for the results.

4. **Second-session infants**: ~~in the main model we include both first- and second-session infants; here we exclude second-session infants.~~ We intended to run an additional analysis where second-session infants were left out. Because there were only 3 such infants, this analysis was not run.

5. **Indistinguishable syllables**: ~~Should there be labs that report that some of the syllables are non-distinguishable in their dominant language in some infants we will exclude those infants here.~~ We intended to run an additional analysis excluding infants where labs report that some of the syllables are non-distinguishable in their dominant language. However, this concern was only raised for a total of 7 infants, so this analysis was not run.

6. **Trial spill-over effects**: In the main model we include trials that are collected after a trial that went missing due to trial-level errors; here we exclude all trials that follow an initial

missing trial due to infant factors as some of the effects may spill-over from the missing trial. This factor was included in the robustness analyses, see the main report for the results.

7. **Missing eye-tracker data**: In the main analysis, trials are included when at least 50% of trial duration has recorded data. ~~Here we will set this percentage to 70, and 90% respectively.~~ We had intended to explore modifying this inclusion percentage. However, we were not able to obtain the data from labs necessary to run this analysis.

Exploratory analyses

Double Familiarization Time

During data collection, a technical error at one lab resulted in familiarization times being twice as long as intended (4 minutes instead of 2); these data were left out of the main analysis. However, this gave us the unique opportunity to exploratorily check whether longer familiarization time would result in rule learning. The fitted model shows a non-significant effect trial_type on looking_time (coefficient=-0.05, p=0.3) and hence there is no evidence of rule learning in this sample. The model results are in Table 2.

Initial Learning?

The second exploratory analysis concerns the question whether the rule-learning effect may be short-lived and only present in the first few trials. Here analysis is limited to the first four trials, and only includes infants that have at least 2 valid trials within those first four trials (as was the case in the main analysis). Table 3 presents the model parameters and accompanying p-values, again clearly indicating that there is no evidence for rule learning.

References

Marcus, G. F., Vijayan, S., Rao, S. B., & Vishton, P. M. (1999). Rule learning by seven-month-old infants. *Science*, *283*(5398), 77–80. https://doi.org/10.1126/science.283.5398.77

Table 2

term	coeff	se	stat	df	p
Intercept	1.80	0.07	27.06	30	0.000
FamRule	-0.04	0.07	-0.65	30	0.519
Type	-0.05	0.05	-1.03	314	0.302
Age	-0.10	0.07	-1.46	30	0.155
Lang	0.06	0.07	0.94	32	0.355
Trial	-0.13	0.02	-5.34	315	0.000
Rep	0.00	0.05	-0.07	314	0.946
Type*Age	-0.07	0.05	-1.31	314	0.191
Type*Lang	-0.01	0.05	-0.24	315	0.807
Type*Trial	0.05	0.05	1.05	315	0.294
Age*Trial	0.05	0.02	1.94	316	0.053

Table 3

Model Estimates for the Data with Trials 1–4 Only Included.

factor	coeff	se	stat	df	p
Intercept	1.51	0.05	28.16	72	0.000
FamRule	-0.03	0.09	-0.40	751	0.690
Type	0.03	0.08	0.46	2079	0.646
Age	-0.02	0.04	-0.59	2364	0.552
Method (CF)	0.11	0.11	1.07	34	0.290
Method (ET)	-0.16	0.09	-1.70	55	0.095
Lang	0.00	0.02	0.15	608	0.877
Trial	-0.23	0.03	-7.54	1776	0.000
Rep	-0.11	0.10	-1.08	1745	0.280
FamRule*Type	-0.12	0.10	-1.16	1745	0.245
Type*Age	0.00	0.02	-0.06	1768	0.955
Type*Method (CF)	-0.04	0.05	-0.69	1765	0.491
Type*Method (ET)	-0.06	0.05	-1.11	1765	0.267
Type*Lang	0.00	0.02	0.15	1761	0.882
Type*Trial	0.04	0.07	0.54	2100	0.590
Age*Trial	-0.03	0.03	-0.92	1783	0.358
Type*Rep	-0.02	0.35	-0.07	760	0.945
